# Sedentary Time and Physical Activity Surveillance Through Accelerometer Pooling in Four European Countries

**DOI:** 10.1007/s40279-016-0658-y

**Published:** 2016-12-10

**Authors:** Anne Loyen, Alexandra M. Clarke-Cornwell, Sigmund A. Anderssen, Maria Hagströmer, Luís B. Sardinha, Kristina Sundquist, Ulf Ekelund, Jostein Steene-Johannessen, Fátima Baptista, Bjørge H. Hansen, Katrien Wijndaele, Søren Brage, Jeroen Lakerveld, Johannes Brug, Hidde P. van der Ploeg

**Affiliations:** 1 Department of Epidemiology and Biostatistics and EMGO+ Institute for Health and Care Research, VU University Medical Center, De Boelelaan 1089a, 1081 HV Amsterdam, The Netherlands; 2 School of Health Sciences, University of Salford, Salford, UK; 3 Department of Sports Medicine, Norwegian School of Sports Sciences, Oslo, Norway; 4 Division of Physiotherapy, Department of Neurobiology, Care Sciences and Society, Karolinska Institutet, Stockholm, Sweden; 5 Exercise and Health Laboratory, CIPER, Faculty of Human Kinetics, Universidade de Lisboa, Lisbon, Portugal; 6 Center for Primary Health Care, IKVMLund University, Malmö, Sweden; 7 Department of Health Sciences, Kristiania University College, Oslo, Norway; 8 MRC Epidemiology Unit, Institute of Metabolic ScienceUniversity of Cambridge, Cambridge, UK; 9 Department of Public and Occupational Health and EMGO Institute for Health and Care Research, VU University Medical Center, Amsterdam, The Netherlands; 10 Sydney School of Public Health, University of Sydney, Sydney, NSW Australia

## Abstract

**Objective:**

The objective of this study was to pool, harmonise and re-analyse national accelerometer data from adults in four European countries in order to describe population levels of sedentary time and physical inactivity.

**Methods:**

Five cross-sectional studies were included from England, Portugal, Norway and Sweden. ActiGraph accelerometer count data were centrally processed using the same algorithms. Multivariable logistic regression analyses were conducted to study the associations of sedentary time and physical inactivity with sex, age, weight status and educational level, in both the pooled sample and the separate study samples.

**Results:**

Data from 9509 participants were used. On average, participants were sedentary for 530 min/day, and accumulated 36 min/day of moderate to vigorous intensity physical activity. Twenty-three percent accumulated more than 10 h of sedentary time/day, and 72% did not meet the physical activity recommendations. Nine percent of all participants were classified as high sedentary and low active. Participants from Norway showed the highest levels of sedentary time, while participants from England were the least physically active. Age and weight status were positively associated with sedentary time and not meeting the physical activity recommendations. Men and higher-educated people were more likely to be highly sedentary, while women and lower-educated people were more likely to be inactive.

**Conclusions:**

We found high levels of sedentary time and physical inactivity in four European countries. Older people and obese people were most likely to display these behaviours and thus deserve special attention in interventions and policy planning. In order to monitor these behaviours, accelerometer-based cross-European surveillance is recommended.

**Electronic supplementary material:**

The online version of this article (doi:10.1007/s40279-016-0658-y) contains supplementary material, which is available to authorized users.

## Key Points

**Table:** 

Accelerometer data showed high levels of sedentary time (530 min/day) and physical inactivity (72% did not meet the physical activity recommendations) in adults in four European countries.
Older people and overweight and obese people are more likely to be highly sedentary and less active, and thus are more at risk for developing certain chronic diseases.
Men and higher-educated people were more likely to be highly sedentary, while women and lower-educated people were more likely to be inactive.

## Introduction

Sedentary behaviour and physical inactivity are well-known risk behaviours for many non-communicable diseases. Sedentary behaviour is defined as any waking behaviour in a sitting or reclining position and a low energy expenditure [1] and is often operationalised as sedentary time or sitting time, while physical inactivity is commonly conceptualised as not meeting the World Health Organization (WHO) physical activity recommendations of 150 min of moderate to vigorous intensity physical activity (MVPA) per week [2]. Both behaviours are associated with increased risk of type 2 diabetes mellitus, cardiovascular disease, certain types of cancer and premature mortality [3–6]. While there is a debate as to whether the associations between sedentary time and health outcomes exist independently of physical activity levels, physical activity is known to attenuate the association between sedentary time and health outcomes [3, 4]. Similarly, a recent study showed that replacing sitting with activity was associated with lower mortality more strongly in inactive than active older adults [7]. These studies show that people who are both highly sedentary and physically inactive might not just be most at risk for the development of non-communicable diseases but might also benefit most from preventive measures.

Within Europe, accurate and comparable data on sedentary time and physical inactivity levels are needed to monitor, compare and benchmark these levels within and across countries, and to target populations at risk. Traditionally, sedentary time and physical activity levels are predominantly assessed by self-reported measures such as questionnaires. However, these measures suffer from limitations such as recall and social desirability bias, limiting their validity [8, 9]. Recently, Steene-Johannessen and colleagues [10] demonstrated low agreement for subjective physical activity data versus objective data when determining adherence to the physical activity recommendations in a European sample, and concluded that self-reported surveillance data should be interpreted with caution. Typically, physical activity time tends to be overestimated and sedentary time tends to be underestimated by self-report. Moreover, in an international context, cultural and linguistic issues in the interpretation of questions or concepts used may hamper comparability between countries and cultures. Objective measures, such as accelerometers, are able to overcome many of these issues and therefore have the potential to provide more accurate and comparable estimates of sedentary time and physical inactivity levels across countries.

Even though there are no cross-European studies that have used accelerometers in population-based samples to date, there are a number of national studies in Europe that have used accelerometers to measure activity behaviour [11]. Because these studies used different algorithms for the interpretation of the accelerometer data (e.g. epoch lengths, non-wear definitions, cut-points for intensity), it is difficult to compare the results based on the published articles. However, such a comparison is possible when the accelerometer count data is harmonised. One of the aims of the DEterminants of DIet and Physical ACtivity (DEDIPAC) knowledge hub is to better utilise existing data by harmonising physical activity and sedentary time surveillance data [12]. Therefore, the aim of this study was to pool, harmonise and re-analyse national accelerometer data in order to estimate and compare levels of sedentary time and physical inactivity across Europe, as well as to assess the associations with several socio-demographic characteristics in order to identify those populations at risk of health-related outcomes.

## Methods

Recently, Wijndaele and colleagues [11] published a systematic literature review reporting on the scope of accelerometer data in adults. In this review, they identified four national population-based studies in European countries that used ActiGraph accelerometers. These studies were from England [13–15], Norway [16], Portugal [17] and Sweden (the ABC [Attitude Behaviour Change] study) [18]. In addition, we included the SNAP (Swedish Neighborhood and Physical Activity) study [19]; even though this study was not based on data from the entire country, it did include a large study sample from the Stockholm area. As this study had similarities with the other studies but was not population based, it served as a case study, providing the opportunity to compare data across countries as well as within Sweden. The principal investigators of the studies were contacted and agreed to participate. Subsequently, data-sharing agreements were signed and the accelerometer count data were transferred to the analysis team.

The characteristics and measurement details of the included studies are shown in Table 1. Four studies were conducted between 2006 and 2009; the Swedish ABC study was conducted in 2001–2002. The size of the analysed samples ranged from 1114 to 3267 across the studies. The studies included varying age groups; we included the age group that was present in the majority of the studies (20–75 years). The reported response rates ranged from 31 to 68%. All studies used random sampling strategies, but they recruited participants in different ways, as shown in Table 1. All studies aimed to include a population-representative sample, with the exception of the Swedish SNAP study. As the primary aim of this study was not population surveillance, it had additional inclusion criteria around how long participants lived in their neighbourhood and whether they were able to walk. All studies used the ActiGraph GT1M (ActiGraph, Pensacola, FL, USA), except for the Swedish ABC study, which used the ActiGraph 7164. All measurements were uniaxial on the vertical acceleration axis. The epoch length varied from 10 to 60 s, which was harmonised to 60 s. The right hip was the most frequent wear site, although participants of the Swedish ABC study wore their accelerometer on the lower back, and participants of the Swedish SNAP study could choose between these two wearing positions. All protocols included 7 consecutive days of wear time, except the Portuguese study, which included a minimum of 4 consecutive days consisting of 2 week days and both weekend days. All participants were asked to wear the accelerometer while they were awake, except during water-based activities (e.g. showering, swimming).

**Table 1:** Study characteristics and measurement details of the included studies

	England	Norway	Portugal (mainland)	Sweden	Sweden (Stockholm)
Study characteristics					
Study name	Health Survey for England [13–15]	N/A [16]	N/A [17]	ABC study [18]	SNAP study [19]
Period of data collection	2008	2008–2009	2006–2008	2001–2002	2008–2009
Sampling strategy	Multi-stage stratified probability design of addresses using the Postcode Address File	Representative sample from the Norwegian population registry	Proportionate stratified random sampling	Randomly selected sample from the Swedish population register	Simple random sampling of individuals from each neighbourhood
Recruitment	Participants were visited at home	Participants received a personalised invitation letter by mail	Participants were recruited from schools, work sites and community settings	Participants were contacted by telephone	Participants received an information letter and were contacted by telephone
Inclusion criteria	Living in a private household in England	Aged between 20 and 85 years	Non-institutionalised people living on the mainland of Portugal	Aged between 18 and 69 years, having a publicly listed telephone number, speaking Swedish and living in Sweden	Living in Sweden since before 2003, having a listed landline or mobile phone number, being able to read and write Swedish, living in the neighbourhood for at least 3 months and having no serious impaired ability to walk
Number of participants	2339 (≥16 years)	3267	1982 (≥18 years)	1114	2269
Age range (years)	≥4	20–85	≥10	18–75	20–66
Reported response rate (%)	58	31	Not reported	68	52
Measurement details					
Monitor type	ActiGraph GT1 M	ActiGraph GT1 M	ActiGraph GT1 M	ActiGraph 7164	ActiGraph GT1 M
Axis	Uniaxial (vertical axis)	Uniaxial (vertical axis)	Uniaxial (vertical axis)	Uniaxial (vertical axis)	Uniaxial (vertical axis)
Wear site	Right hip	Right hip	Right hip	Lower back	Hip or lower back
Protocol wear time	7 consecutive days	7 consecutive days	At least 4 consecutive days (2 week days and 2 weekend days)	7 consecutive days	7 consecutive days
Protocol non-wear	While asleep and during water-based activities	While asleep and during water-based activities	While asleep and during water-based activities	While asleep and during water-based activities	While asleep and during water-based activities
Epoch length (s)	60	10	15	60	60

*N/A* not applicable

### Accelerometer Data

We used ActiLife (version 6.12.0; ActiGraph) to convert DAT-files to AGD-files and reintegrate files to 60 s epoch length where appropriate. STATA^®^ (version 12.1; STATACorp LP, College Station, TX, USA) was used for the wear time validation and intensity classification. We excluded any spurious data points defined as >20,000 counts/min. Non-wear time was defined as bouts of ≥60 min of consecutive zero counts, allowing interruptions of up to two non-zero counts (≤100 counts/min) [20]. A valid day was defined as a minimum of 600 wear time min/day and participants needed a minimum of 4 valid days to be included in the analyses; we did not discriminate between week and weekend days. We used the Troiano cut-points to define sedentary time (<100 counts/min), and light- (100–2019 counts/min), moderate- (2020–5998 counts/min) and vigorous-intensity physical activity (≥5999 counts/min) [20]. The number of and time spent in consecutive sedentary bouts of ≥30 and ≥60 min were calculated. In addition, the number of and time spent in MVPA bouts of ≥10 min were calculated, allowing for up to 2 min below the MVPA threshold [20].

In SPSS^®^ (version 22; IBM Corp., Armonk, NY, USA), day-to-day data were aggregated to mean values per day using all valid days. Variables were computed to indicate whether participants met the physical activity recommendations of ≥150 min of MVPA/week (defined as ≥21.42 min of MVPA/mean day, not discriminating between moderate or vigorous physical activity), based on the total time in MVPA and time in MVPA bouts of ≥10 min. We used these two definitions because several recommendations, including the WHO recommendations, state that the activity should occur in bouts of at least 10 min [2], while others, such as the Australian guidelines, do not include these bouts [21]. Furthermore, participants were classified according to whether they accumulated more than 7.5 and 10 h of sedentary time/day. The cut-point of 7.5 sedentary h/day was based on a meta-analysis suggesting that the risk for all-cause mortality risk increases between 7 and 8 h of sedentary time/day [4]. However, because this research was mostly based on self-reported measures, and sedentary time tends to be underreported, we selected an additional cut-point of 10 h based on validation studies from the Health Survey for England and the Norwegian study showing that participants on average reported between 2 and 2.5 h less sedentary time than accelerometer data [22, 23]. Finally, we classified participants as being high sedentary/low active if they accumulated more than 10 h of sedentary time/day and did not meet the physical activity recommendations based on total time in MVPA, which is considered the group at highest risk to develop non-communicable diseases.

### Socio-Demographic Characteristics

In addition to the accelerometer data, four socio-demographic characteristics were assessed in all studies and could hence be harmonised: sex, age, weight status based on body mass index (BMI) and educational level. All variables were self-reported, except for height and weight (used to calculate BMI) in the Health Survey for England and the Portuguese study, which were objectively measured. We included age in four categories: 20–35, 36–50, 51–66 and 67–75 years. The cut-off of the oldest category was based on the study sample of the Swedish SNAP study, since they only included participants up to 66 years old. By using this cut-off, the oldest category did not comprise any participants from the Swedish SNAP study. We categorised BMI into normal weight (including underweight; BMI <25 kg/m^2^), overweight (BMI 25–30 kg/m^2^) and obese (BMI ≥30 kg/m^2^) according to WHO guidelines [24]. There was variation in the assessment of educational level. The Health Survey for England used respondents’ age at which they finished full-time education, the Portuguese study used the number of years people attended education, while the three remaining studies used the level of the highest completed education (with different classifications across studies). Since direct mapping of the categories from these different variables was not possible, we categorised this variable into quartiles at the study level to provide a rough estimate of education level.

### Statistical Analyses

All analyses were conducted in SPSS^®^ (version 22). Descriptive statistics were used to assess sample characteristics as well as levels of sedentary time and physical activity. In addition, multivariable logistic regression analyses were conducted to obtain the odds ratios (ORs) of (1) sitting more than 7.5 h/day; (2) sitting more than 10 h/day; (3) meeting the physical activity recommendations based on total time in MVPA; (4) meeting the physical activity recommendations using MVPA time in bouts of ≥10 min; and (5) being classified as high sedentary/low active. These analyses were conducted on the total sample and stratified by study sample. The overall analyses were adjusted for within-study correlations by adding dummy variables for study. Since we only had a small number of studies, this results in a more valid estimation of the variance than multilevel analyses. Sex, age, weight status and level of education were included as independent variables. In addition, wear time was included as a covariate. Statistical significance was set at *p* < 0.05.

## Results

We obtained accelerometer data from 12,071 participants, 9509 of which were aged 20–75 years and had >4 valid days, and were thus included in analyses. The number of participants per study ranged from 1059 in the Swedish ABC study to 3098 in the Norwegian study. Of the participants, 56% were female, with a mean age of 48 years and a mean BMI of 26 kg/m^2^. When combining the study-specific quartiles for educational level, 17% of the participants belonged to the lowest category, followed by 26, 22 and 35% in the other three categories. All sample characteristics of the total sample and the separate study samples are shown in Table 2.

**Table 2:** Socio-demographic sample characteristics of the total sample and the separate study samples

Characteristics	Total	England	Norway	Portugal	Sweden	
					ABC study	SNAP study
*n* (% of total)	9509 (100)	1799 (18.9)	3098 (32.6)	1183 (12.4)	1059 (11.1)	2370 (24.9)
Sex						
Women [*n* (%)]	5273 (55.5)	978 (54.4)	1656 (53.5)	741 (62.6)	589 (55.6)	1309 (55.2)
Age (years)						
Mean (SD)	47.8 (14.1)	50.2 (15.0)	47.9 (13.7)	48.9 (17.1)	45.9 (14.3)	46.0 (11.8)
20–35 [*n* (%)]	2133 (22.4)	369 (20.5)	657 (21.2)	318 (26.9)	297 (28.0)	492 (20.8)
36–50 [*n* (%)]	3178 (33.4)	481 (26.7)	1080 (34.9)	332 (28.1)	328 (31.0)	957 (40.4)
51–66 [*n* (%)]	3252 (34.2)	655 (36.4)	1074 (34.7)	263 (22.2)	339 (32.0)	921 (38.9)
67–75 [*n* (%)]	946 (9.9)	294 (16.3)	287 (9.3)	270 (22.8)	95 (9.0)	N/A
Weight status						
Mean BMI [kg/m^2^ (SD)]	25.7 (4.3)	27.7 (5.2)	25.5 (4.0)	26.4 (4.0)	25.1 (3.6)	24.6 (3.7)
Normal (BMI <25) [*n* (%)]	4474 (49.2)	522 (31.5)	1517 (50.9)	442 (40.7)	560 (54.7)	1433 (61.4)
Overweight (BMI 25–30) [*n* (%)]	3310 (36.4)	676 (40.7)	1105 (37.0)	448 (41.3)	369 (36.0)	712 (30.5)
Obese (BMI ≥30) [*n* (%)]	1302 (14.3)	461 (27.8)	361 (12.1)	196 (18.0)	95 (9.3)	189 (8.1)
Education [*n* (%)]						
Lowest	1545 (17.0)	510 (28.3)	381 (12.4)	202 (25.5)	215 (20.4)	237 (10.0)
Second lowest	2358 (25.9)	501 (27.8)	1153 (37.4)	150 (18.9)	244 (23.1)	310 (13.1)
Second highest	1990 (21.9)	308 (17.1)	739 (24.0)	149 (18.8)	253 (24.0)	541 (22.8)
Highest	3203 (35.2)	480 (26.7)	808 (26.2)	292 (36.8)	343 (32.5)	1280 (54.1)

*BMI* body mass index, *N/A* not applicable, *SD* standard deviation

Table 3 summarises the sedentary time and physical activity outcomes. In general, the accelerometers were worn for 6–7 days except in Portugal, where the mean number of valid days was 4.8. Mean wear time was 14.5 h/day. Of the daily wear time, 61% was spent sedentary and only 2 min on vigorous-intensity physical activities. Averaging all valid days, participants accumulated a mean of 133 min in 2.8 (median) sedentary bouts of ≥30 min, and a mean of 31 min in 0.3 (median) sedentary bouts of ≥60 min/day. Furthermore, on average, 16 min/day were spent in 0.6 (median) MVPA bouts of ≥10 min. These low numbers for sedentary time and MVPA are explained by the fact that most participants did not accumulate ≥60 min of sedentary time or ≥10 min of MVPA on most days. Eighty percent of the participants were sedentary for more than 7.5 h/day, and almost one-quarter were sedentary for more than 10 h/day. One-third of the participants did not meet the physical activity recommendations based on total time in MVPA, while more than 70% did not meet the recommendations based on time in MVPA bouts of ≥10 min. We also calculated the percentage of people meeting the recommendations by 150 min of MVPA/week, 75 min of vigorous-intensity physical activity (VPA)/week, or an equivalent combination, which resulted in a 1% increase (results not shown). Finally, 9% of the participants were classified as low sedentary/high active. An overview of these outcomes stratified by sex, age, weight status and educational level is provided in Electronic Supplementary Material Table S1.

**Table 3:** Accelerometer-assessed sedentary time and physical activity in the total sample and the separate study samples

	Total	England	Norway	Portugal	Sweden	
					ABC study	SNAP study
Mean (SD) number of valid days	6.43 (1.15)	6.45 (0.87)	6.87 (0.91)	4.77 (1.17)	6.82 (1.08)	6.50 (0.88)
Mean (SD) min/day wear time	869.57 (72.62)	849.33 (71.93)	891.26 (66.56)	839.51 (75.83)	871.44 (70.58)	870.78 (70.52)
Mean (SD) activity kcounts/day	305.60 (127.36)	276.36 (129.42)	305.99 (123.66)	300.27 (129.19)	314.23 (125.14)	326.09 (126.36)
Mean (SD) min/day sed time	530.13 (91.88)	519.58 (91.69)	553.00 (83.04)	485.45 (96.9)	497.69 (91.97)	545.06 (86.97)
Mean (SD) min/day light PA	303.81 (83.16)	300.07 (83.56)	303.02 (79.11)	321.09 (91.35)	340.91 (87.32)	282.50 (74.01)
Mean (SD) min/day moderate PA	33.48 (22.43)	28.46 (23.09)	32.88 (21.29)	31.48 (22.56)	31.13 (21.17)	40.12 (22.34)
Mean (SD) min/day vigorous PA	2.15 (5.77)	1.22 (4.13)	2.37 (6.02)	1.49 (5.15)	1.70 (4.37)	3.11 (7.05)
Mean (SD) min/day MVPA	35.63 (24.36)	29.68 (24.39)	35.24 (23.44)	32.97 (24.15)	32.83 (22.68)	43.23 (24.49)
Percentage (SD) sed of wear time	61.00 (9.48)	61.25 (9.98)	62.13 (8.63)	57.90 (10.68)	57.18 (9.77)	62.58 (8.55)
Percentage (SD) light PA of wear time	34.92 (9.07)	35.28 (9.17)	33.93 (8.24)	38.20 (10.21)	39.07 (9.22)	32.45 (8.17)
Percentage (SD) moderate PA of wear time	3.84 (2.53)	3.33 (2.65)	3.68 (2.38)	3.73 (2.65)	3.56 (2.37)	4.61 (2.48)
Percentage (SD) vigorous PA of wear time	0.25 (0.66)	0.14 (0.47)	0.26 (0.67)	0.18 (0.62)	0.19 (0.50)	0.36 (0.82)
Percentage (SD) MVPA of wear time	4.09 (2.75)	3.47 (2.80)	3.94 (2.61)	3.91 (2.84)	3.76 (2.54)	4.97 (2.74)
Median (IQR) number ≥30 min sed bouts	2.75 (1.83–3.86)	2.71 (1.71–3.86)	2.86 (2.00–4.00)	2.50 (1.60–3.50)	2.43 (1.57–3.38)	2.94 (2.00–4.00)
Mean (SD) min/day ≥30 min sed bouts	132.94 (72.60)	130.57 (75.02)	139.59 (71.91)	120.86 (69.75)	118.61 (72.41)	138.48 (71.37)
Median (IQR) number ≥60 min sed bouts	0.29 (0.14–0.57)	0.29 (0.00–0.50)	0.29 (0.14–0.57)	0.25 (0.00–0.50)	0.29 (0.14–0.50)	0.29(0.14–0.57)
Mean (SD) min/day ≥60 min sed bouts	31.17 (34.14)	28.26 (32.11)	33.57 (33.79)	29.80 (34.79)	30.61 (40.88)	31.18 (32.20)
Median (IQR) number ≥10 min MVPA bouts^a^	0.57 (0.14–1.17)	0.40 (0.00–1.00)	0.57 (0.17–1.14)	0.50 (0.00–1.00)	0.43 (0.14–0.86)	0.86 (0.33–1.50)
Mean (SD) min/day ≥10 min MVPA bouts^a^	15.90 (18.18)	11.81 (16.77)	17.79 (19.27)	12.83 (16.82)	12.10 (14.48)	19.76 (18.69)
Percentage >7.5 h/day sed	81.1	77.8	88.7	66.7	69.5	86.0
Percentage >10 h/day sed	22.8	18.8	29.2	12.1	14.0	26.7
Percentage not meeting PA recs—based on total time in MVPA	31.8	45.7	31.1	37.6	36.3	17.5
Percentage not meeting PA recs—based on time in ≥10 min MVPA bouts^a^	72.1	81.3	68.4	79.4	79.3	63.1
Percentage >10 h/day sed and not meeting PA recs based on total MVPA	8.7	11.3	11.3	5.2	7.1	5.9

*IQR* interquartile range, *kcounts* kilocounts, *MVPA* moderate to vigorous physical activity, *PA* physical activity, *recs* recommendations, *SD* standard deviation, *sed* sedentary

^a^With allowance for interruptions of maximum 2 min below threshold

**Fig. 1:**
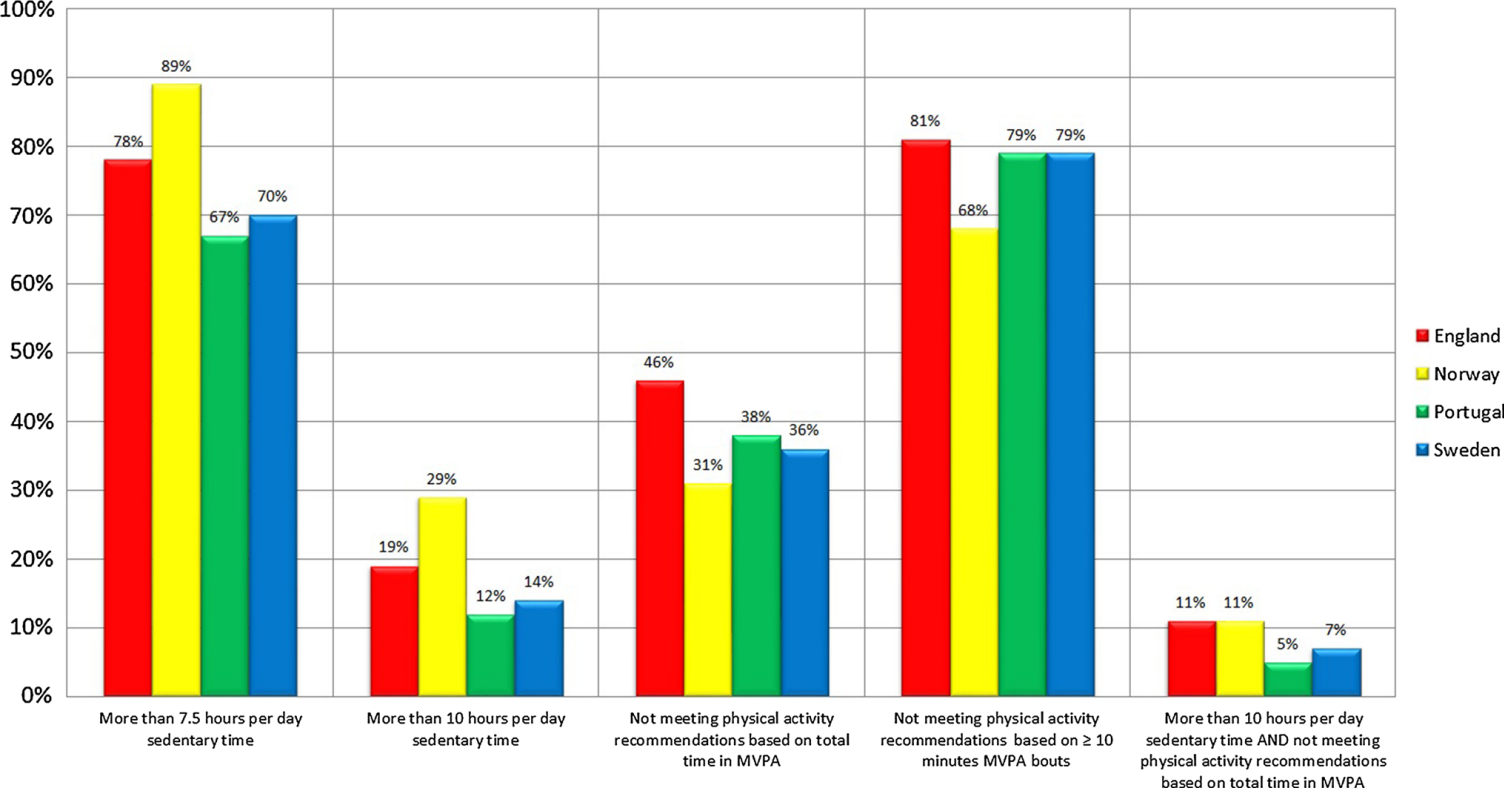
Sedentary time and physical inactivity in England, Norway, Portugal and Sweden. This figure is based on the four national population-based studies included in this research and shows the percentage of participants accumulating more than 7.5 and 10 h of sedentary time/day, the percentage of participants not meeting the physical activity recommendations based on total time in MVPA and time in ≥10 min MVPA bouts, and the percentage of participants accumulating more than 10 h of sedentary time per day and not meeting the physical activity recommendation based on total time in MVPA across the different countries. *MVPA* moderate to vigorous physical activity

Figure 1 further visualises the country-specific differences based on the four national population-based studies. Participants from Norway consistently showed the highest levels of sedentary time, followed by participants from England, Sweden and Portugal. Participants from England showed the highest percentages of not meeting (both) physical activity recommendations, followed by participants from Portugal, Sweden and Norway. With regards to the high sedentary/low active classification, participants from Norway and England showed higher percentages (11%) than those from Sweden (7%) and Portugal (5%).

The multivariable ORs of accumulating more than 10 h of sedentary time/day are shown in Table 4 for the total sample as well as for the separate study samples. In the total sample, men, people aged 67–75 years, obese people, and higher-educated people had a significantly higher OR of sitting more than 10 h/day. The direction of these associations was the same in the majority of the separate study samples, with varying levels of significance. Similar analyses using 7.5 h/day as a cut-point are summarised in Electronic Supplementary Material Table S2 and showed reasonably similar results.

**Table 4:** Distribution and multivariable odds ratio of accumulating more than 10 h/day of sedentary time by sex, age, body mass index and educational level

	Total		England		Norway		Portugal		Sweden			
									ABC study		SNAP study	
	%	OR (95% CI)^a^	%	OR (95% CI)^b^	%	OR (95% CI)^b^	%	OR (95% CI)^b^	%	OR (95% CI)^b^	%	OR (95% CI)^b^
Overall	22.8	N/A	18.8	N/A	29.2	N/A	12.1	N/A	14.0	N/A	26.7	N/A
Sex												
Men (ref)	31.0	1.00	27.4	1.00	37.7	1.00	15.4	1.00	18.3	1.00	36.9	1.00
Women	16.2	**0.47 (0.42–0.52)**	11.7	**0.39 (0.29–0.51)**	21.8	**0.49 (0.41–0.58)**	10.1	0.63 (0.39–1.00)	10.5	**0.64 (0.43–0.95)**	18.5	**0.41 (0.34–0.51)**
Age (years)												
20–35 (ref)	21.7	1.00	17.1	1.00	27.7	1.00	13.2	1.00	13.8	1.00	27.2	1.00
36–50	22.2	**0.77 (0.66–0.90)**	13.3	0.73 (0.47–1.14)	29.4	0.90 (0.71–1.14)	12.3	0.92 (0.50–1.69)	14.3	0.97 (0.59–1.61)	24.5	**0.57 (0.43–0.76)**
51–66	24.4	0.96 (0.82–1.12)	20.9	1.06 (0.70–1.60)	29.9	1.00 (0.78–1.27)	10.3	1.13 (0.54–2.36)	13.3	1.10 (0.66–1.86)	28.8	0.80 (0.60–1.08)
67–75	21.8	**2.01 (1.59–2.54)**	25.5	**2.44 (1.49–3.98)**	28.9	**1.76 (1.23–2.50)**	12.2	**3.57 (1.53–8.33)**	15.8	2.04 (0.99–4.22)	N/A	N/A
Weight status												
BMI <25 (ref)	22.1	1.00	16.1	1.00	26.7	1.00	12.2	1.00	14.5	1.00	25.3	1.00
BMI 25–30	23.5	1.06 (0.93–1.20)	17.9	0.91 (0.64–1.28)	31.5	1.17 (0.97–1.42)	12.3	0.99 (0.58–1.69)	13.3	0.83 (0.55–1.28)	28.9	1.07 (0.85–1.35)
BMI ≥30	25.3	**1.58 (1.33–1.87)**	22.8	**1.63 (1.13–2.35)**	35.7	**1.61 (1.22–2.11)**	13.3	1.44 (0.70–2.95)	14.7	1.23 (0.63–2.40)	29.6	**1.58 (1.07–2.32)**
Education												
Lowest (ref)	18.2	1.00	19.4	1.00	22.6	1.00	9.9	1.00	9.3	1.00	23.6	1.00
Second lowest	19.0	0.86 (0.71–1.05)	14.2	0.75 (0.50–1.12)	23.4	0.97 (0.71–1.32)	6.0	0.82 (0.33–2.09)	12.3	1.17 (0.60–2.25)	21.9	0.98 (0.61–1.58)
Second highest	25.2	**1.46 (1.20–1.78)**	14.9	0.87 (0.55–1.38)	33.3	**1.87 (1.36–2.57)**	15.4	2.12 (0.89–5.00)	16.2	1.80 (0.95–3.42)	27.0	1.27 (0.83–1.95)
Highest	27.7	**1.81 (1.50–2.17)**	25.6	**1.91 (1.29–2.83)**	36.9	**2.19 (1.60–3.01)**	16.4	**2.96 (1.28–6.83)**	16.0	1.67 (0.91–3.05)	28.3	**1.56 (1.05–2.30)**

Results are shown for the total sample and the different study samples. Numbers in bold represent *p* < 0.05

*BMI* body mass index, *CI* confidence interval, *N/A* not applicable, *OR* odds ratio, *ref* reference

^a^Adjusted for sex, age, BMI, educational level, study and wear time

^b^Adjusted for sex, age, BMI, educational level and wear time

Table 5 shows the multivariable ORs of not meeting the physical activity recommendations based on total time in MVPA. Women, older people, overweight and obese people, and lower-educated people showed significantly higher ORs of not meeting the physical activity recommendations in the total sample. ORs increased with increasing age and weight status. While the levels of significance differed across separate study samples, the directions of association were consistent across the majority of the studies. Electronic Supplementary Material Table S3 shows similar analyses using time spent in MVPA bouts of ≥10 min. While the findings were similar for weight status and educational level, the associations with sex and age were less clear.

**Table 5:** Distribution and multivariable odds ratio of not meeting physical activity recommendations based on accumulating more than 150 min/week of moderate to vigorous physical activity by sex, age, body mass index and educational level

	Total		England		Norway		Portugal		Sweden			
									ABC study		SNAP study	
	%	OR (95% CI)^a^	%	OR (95% CI)^b^	%	OR (95% CI)^b^	%	OR (95% CI)^b^	%	OR (95% CI)^b^	%	OR (95% CI)^b^
Overall	31.8	N/A	45.7	N/A	31.1	N/A	37.6	N/A	36.3	N/A	17.5	N/A
Sex												
Men (ref)	27.6	1.00	37.4	1.00	28.5	1.00	28.3	1.00	31.9	1.00	16.6	1.00
Women	35.3	**1.51 (1.37–1.67)**	52.7	**2.01 (1.63–2.49)**	33.3	**1.39 (1.18–1.64)**	43.2	**2.03 (1.47–2.80)**	39.7	**1.48 (1.12–1.97)**	18.2	1.22 (0.97–1.53)
Age (years)												
20–35 (ref)	25.3	1.00	35.2	1.00	28.8	1.00	30.8	1.00	22.6	1.00	11.4	1.00
36–50	26.0	1.07 (0.93–1.23)	33.1	0.85 (0.62–1.17)	28.7	0.99 (0.79–1.25)	29.2	0.95 (0.63–1.45)	34.5	**2.01 (1.36–2.96)**	15.4	1.44 (1.01–2.04)
51–66	34.7	**1.37 (1.19–1.57)**	48.5	**1.42 (1.04–1.93)**	32.1	1.03 (0.81–1.29)	38.4	1.05 (0.63–1.75)	44.8	**2.55 (1.72–3.78)**	22.9	**2.15 (1.53–3.03)**
67–75	56.6	**2.07 (1.71–2.52)**	73.1	**3.90 (2.64–5.76)**	41.5	1.19 (0.86–1.63)	55.2	1.73 (0.96–3.09)	54.7	**3.69 (2.14–6.37)**	N/A	N/A
Weight status												
BMI <25 (ref)	24.1	1.00	37.7	1.00	25.4	1.00	32.6	1.00	28.4	1.00	13.5	1.00
BMI 25–30	33.1	**1.39 (1.25–1.55)**	41.4	1.20 (0.93–1.54)	33.7	**1.55 (1.29–1.85)**	33.7	0.85 (0.59–1.23)	41.2	**1.57 (1.16–2.12)**	19.7	**1.45 (1.13–1.86)**
BMI ≥30	52.2	**2.78 (2.41–3.21)**	59.0	**2.35 (1.78–3.11)**	48.2	**2.77 (2.16–3.55)**	51.5	**2.04 (1.28–3.27)**	63.2	**3.99 (2.46–6.49)**	38.6	**3.71 (2.64–5.22)**
Education												
Lowest (ref)	48.0	1.00	57.6	1.00	44.4	1.00	47.5	1.00	52.1	1.00	29.5	1.00
Second lowest	34.7	**0.76 (0.65–0.88)**	43.1	0.75 (0.56–1.01)	34.5	**0.73 (0.56–0.94)**	34.0	0.83 (0.50–1.38)	38.9	0.90 (0.59–1.37)	19.0	**0.54 (0.35–0.82)**
Second highest	30.3	**0.78 (0.66–0.91)**	45.1	1.02 (0.73–1.42)	30.4	**0.64 (0.48–0.84)**	34.2	0.97 (0.56–1.68)	32.8	0.84 (0.55–1.30)	19.4	0.74 (0.51–1.08)
Highest	22.0	**0.56 (0.48–0.65)**	36.0	0.76 (0.56–1.04)	20.4	**0.39 (0.29–0.52)**	32.2	0.97 (0.57–1.65)	27.1	**0.52 (0.35–0.77)**	14.1	**0.48 (0.34–0.68)**

Results are shown for the total sample and the different study samples. Numbers in bold represent *p* < 0.05

*BMI* body mass index, *CI* confidence interval, *N/A* not applicable, *OR* odds ratio, *ref* reference

^a^Adjusted for sex, age, BMI, educational level, study and wear time

^b^Adjusted for sex, age, BMI, educational level and wear time

Finally, the ORs of being classified as high sedentary/low active are shown in Table 6. Men, older people, and overweight and obese people showed significantly higher ORs of being high sedentary/low active. No clear pattern with educational level was observed. These associations were comparable in the majority of the separate study samples, with varying levels of significance.

**Table 6:** Distribution and multivariable odds ratio of accumulating more than 10 h/day of sedentary time and not meeting physical activity recommendations based on accumulating more than 150 min/week of moderate to vigorous physical activity by sex, age, body mass index and educational level

	Total		England		Norway		Portugal		Sweden			
									ABC study		SNAP study	
	%	OR (95% CI)^a^	%	OR (95% CI)^b^	%	OR (95% CI)^b^	%	OR (95% CI)^b^	%	OR (95% CI)^b^	%	OR (95% CI)^b^
Overall	8.7	N/A	11.3	N/A	11.3	N/A	5.2	N/A	7.1	N/A	5.9	N/A
Sex												
Men (ref)	11.0	1.00	15.1	1.00	13.5	1.00	6.1	1.00	8.3	1.00	7.6	1.00
Women	6.9	**0.73 (0.62–0.85)**	8.2	**0.55 (0.39–0.77)**	9.4	**0.78 (0.62–0.99)**	4.7	0.87 (0.46–1.66)	6.1	0.94 (0.57–1.57)	4.4	**0.69 (0.48–1.00)**
Age (years)												
20–35 (ref)	6.0	1.00	6.2	1.00	9.9	1.00	3.5	1.00	4.0	1.00	3.3	1.00
36–50	6.7	0.97 (0.76–1.23)	5.2	0.77 (0.41–1.45)	10.4	0.94 (0.67–1.31)	5.7	1.76 (0.74–4.22)	7.9	**2.27 (1.07–4.82)**	3.3	0.80 (0.42–1.50)
51–66	10.4	**1.47 (1.17–1.85)**	13.6	1.71 (0.99–2.95)	11.7	1.06 (0.76–1.48)	3.4	1.24 (0.41–3.76)	7.1	2.17 (0.99–4.77)	9.9	**2.50 (1.42–4.40)**
67–75	15.8	**3.17 (2.37–4.24)**	22.8	**4.97 (2.72–9.07)**	16.0	**1.91 (1.23–2.95)**	8.5	**4.45 (1.42–13.97)**	13.7	**6.32 (2.54–15.72)**	N/A	N/A
Weight status												
BMI <25 (ref)	6.3	1.00	7.7	1.00	8.5	1.00	4.5	1.00	6.3	1.00	4.1	1.00
BMI 25–30	9.6	**1.37 (1.15–1.64)**	9.9	0.98 (0.63–1.52)	12.9	**1.50 (1.16–1.95)**	5.8	1.12 (0.52–2.39)	7.6	1.09 (0.63–1.89)	7.9	**1.71 (1.16–2.54)**
BMI ≥30	14.7	**2.46 (1.99–3.05)**	16.1	**2.00 (1.29–3.11)**	19.1	**2.46 (1.77–3.42)**	7.1	2.12 (0.88–5.09)	11.6	**2.21 (1.05–4.66)**	12.7	**3.34 (1.97–5.66)**
Education												
Lowest (ref)	12.0	1.00	15.9	1.00	13.4	1.00	7.9	1.00	6.5	1.00	9.7	1.00
Second lowest	8.3	**0.72 (0.57–0.91)**	8.6	0.63 (0.39–1.01)	10.1	0.76 (0.52–1.09)	1.3	0.25 (0.05–1.20)	7.4	1.22 (0.56–2.65)	4.8	0.58 (0.29–1.17)
Second highest	9.5	1.08 (0.84–1.37)	8.4	0.82 (0.48–1.40)	13.8	1.16 (0.79–1.71)	6.7	1.45 (0.50–4.16)	8.3	1.75 (0.81–3.76)	5.5	0.80 (0.44–1.46)
Highest	7.6	0.94 (0.75–1.19)	11.3	1.21 (0.76–1.91)	9.7	0.79 (0.53–1.18)	6.2	1.63 (0.58–4.63)	6.1	0.99 (0.47–2.11)	5.5	0.83 (0.49–1.39)

Results are shown for the total sample and the different study samples. Numbers in bold represent *p* < 0.05

*BMI* body mass index, *CI* confidence interval, *N/A* not applicable, *OR* odds ratio, *ref* reference

^a^Adjusted for sex, age, BMI, educational level, study and wear time

^b^Adjusted for sex, age, BMI, educational level and wear time

## Discussion

Our findings indicate that participants on average accumulated 8–9 h of sedentary time/day and that 80% accumulated at least 7.5 sedentary h/day. These numbers are similar to the results of the 2005–2006 National Health and Nutrition Examination Survey (NHANES) accelerometer study from the USA [25] that reported a mean of 8 sedentary h/day. The current estimates are much higher than previous European studies based on questionnaires, which reported a median of 5 h of sedentary time/day [26–28] and that 20% were sedentary ≥7.5 h/day [28, 29]. Interestingly, we found that approximately 20% of the participants were sedentary ≥10 h/day. The largest difference between the most recent Eurobarometer survey and the current study can be found in Portugal, with the Eurobarometer reporting 5 h less sedentary time (180 min/day) than the present objective assessments of 485 min/day [28].

With regard to physical activity, participants accumulated a mean of 36 min of MVPA/day; however, 32% did not meet the physical activity recommendations of ≥150 min/week based on total time in MVPA. This latter finding is similar to a study by Hallal and colleagues [30], which relied on self-report questionnaires and concluded that 35% of European adults did not meet the physical activity recommendations defined as ≥30 min of MVPA on 5 days/week, ≥20 min of VPA on 3 days/week or an equivalent combination. However, when we calculated these percentages based on the time in MVPA bouts of ≥10 min, as is currently recommended by the WHO [2], over 70% of the participants did not meet the physical activity recommendations. Even though this is a substantial difference, these numbers are still lower than the percentages reported in the 2003–2004 NHANES accelerometer study from the USA, which reported that approximately 97% of the participants did not meet the physical activity recommendations when taking into account similar ≥10 min MVPA bouts [20]. It should be noted, however, that they defined this as accumulating ≥30 min of MVPA on 5 days/week, which is slightly different from our definition of ≥150 min of MVPA/week.

These findings support the notion that prevalence data based on subjective measures substantially underestimates sedentary time and overestimates physical activity. The interpretation of these findings is somewhat difficult, as the physical activity recommendations are largely based on self-reported studies using questionnaires when examining associations between activity and health-related outcomes. This might mean that the questionnaire-induced overestimation of physical activity levels is already taken into account in the recommendations and that the recommendations may change based on future large-scale longitudinal studies using objective measurements related to health-related outcomes. Although there are no specific public health recommendations regarding sedentary time, most epidemiological evidence regarding the health risks of sedentary behaviours is also based on self-reported data. Hence, the translation of accelerometer-based surveillance data with regard to public health risks is not straightforward. However, to date, few studies have been performed on the association between objectively measured physical activity and/or sedentary time and clinical health-related outcomes.

Of the population-based studies included in our analyses, Norway had the highest levels of sedentary time and the lowest percentage of not meeting physical activity recommendations, which further emphasises that these behaviours are not the inverse of each other and may coexist. England was the least physically active country, and both England and Norway showed relatively high percentages of people being high sedentary/low active. The relative order of the countries in our pooled analysis is roughly the same as in previous research, with Norway and Sweden generally more sedentary but also more physically active than England and Portugal [26, 28, 30, 31]. Even though our study included northern and southern European countries, the limited amount and geographical dispersion of included countries calls for more research to assess the wider distribution of sedentary time and physical activity across Europe.

The Swedish SNAP study showed substantially lower levels of physical inactivity and higher levels of sedentary time than the Swedish ABC study. The ABC study was set up as a population-based surveillance study for Sweden, while the SNAP study was not designed or primarily aimed at population surveillance but rather to study environmental correlates in the Stockholm metropolitan area. When looking at the characteristics of the two Swedish study samples, the SNAP study has a larger proportion of higher-educated people. Based on our findings that higher-educated people are more sedentary and less physically inactive, it might be that the SNAP study overestimated the levels of sedentary time and underestimated the levels of physical inactivity in Sweden. Having said that, the ABC study was conducted earlier (in 2001–2002), using an older model accelerometer (ActiGraph 7164) and had a different wear site (the lower back), while the SNAP study was more comparable to the other studies in this respect. However, a sub-sample of the ABC study were followed-up in 2007–2008 (500 participants) and although sedentary time had increased by half an hour per day, the overall results were similar [32]. Even though studies have shown that there are no significant differences between accelerometer placement on the hip or lower back [33], there is some discussion about the comparability of the results of the two ActiGraph models [34–36]. In conclusion, the marked difference between the two studies illustrates the importance of using an appropriate study sample for accurate population surveillance of sedentary time and physical activity.

Being in the highest age group (67–75 years) and being obese was consistently associated with more sedentary time, less physical activity and being classified as high sedentary/low active. The strength of the association increased with increasing age and weight status for not meeting the physical activity recommendations and being classified as high sedentary/low active (although not for being sedentary for more than 10 h/day). These findings are in line with previous findings reported in reviews of the correlates of sedentary behaviour [37] and physical activity [38] and indicate that populations that are older and have a higher BMI need special attention in interventions and policies aiming to improve these lifestyle behaviours. It should be noted, however, that the relative intensity of activities is different for older adults, with research showing cut-points around <22 counts/min for sedentary behaviour [39] and ≥1040 counts/min for MVPA [40]. This means that we might have overestimated sedentary time and physical inactivity in this group using the regular cut-points (of <100 and ≥2020 counts/min, respectively).

Men and higher-educated people were more likely to be highly sedentary, but also more likely to meet the physical activity recommendations. This is in line with previous research reported in systematic reviews [37, 38]. Instead of sedentary time and moderate-intensity activities, women accumulated more time in light-intensity physical activities than men. Higher-educated people might be more likely to have a desk job, and hence accumulate a lot of sedentary time/day, while they might also be more inclined to exercise and therefore are more likely to meet the physical activity recommendations. Men were more likely to be classified as high sedentary/low active than women, while education did not show a clear association with this classification. It should be noted, however, that we have used a crude way of categorising education, since it was difficult to harmonise between studies. Overall, public health efforts aiming to enhance these lifestyle behaviours should pay special attention to increasing physical activity in women and lower-educated people, and to decreasing sedentary time in men and higher-educated people. In men, specifically, extra attention could be given to decrease the combination of high sedentary time and low physical activity.

Across all studies, 8.5% of the participants were classified as high sedentary/low active because they accumulated more than 10 h of sedentary time/day and did not meet the physical activity recommendations based on total time in MVPA. As this phenomenon has not been studied extensively before, and the prevalence numbers are highly dependent on the way the variable is defined, it is difficult to draw comparisons with other studies. However, as these people might be most at risk for adverse health outcomes, more research targeting this group is warranted.

In order to assess the added value of pooled analyses, we compared the ranking of the countries based on the original country-specific articles with the ranking based on our pooled results. For MVPA min/day, the order of most active countries based on the original articles was (1) Norway, (2) Sweden, (3) England and (4) Portugal, while this was (1) Norway, (2) Portugal, (3) Sweden and (4) England based on our analysis. For sedentary min/day, the ranking was (1) England, (2) Portugal, (3) Norway and (4) Sweden based on the original articles, while this was (1) Norway, (2) England, (3) Sweden and (4) Portugal based on the pooled data. This was the case for the total sample as well as for the stratified samples by sex. These differences are caused by methodological differences across the original studies (e.g. non-wear definitions, intensity cut-points) and illustrate the importance of harmonisation and standardisation. Even though studies seem reasonably similar at first glance, small but significant differences in methodology may cause large differences in results and consequently the conclusions. This can only be solved by a priori universal standardisation or post-priori pooling, harmonising and re-analysing the accelerometer count data, applying the exact same algorithms to all data involved.

### Strengths and Limitations

This study is the first to combine population-based accelerometer data from all four European countries that have collected such data in adults. The main strength of this study is that accelerometer count data were centrally pooled, harmonised and re-analysed, using identical definitions for the interpretation of the accelerometer data (e.g. epoch lengths, cut-points) across studies.

A limitation of this study is the possible differences in sampling methodology across studies. Even though four of the studies were set up as national population-based studies, it is unclear how representative the studies really are of the population of their country. The reported response rate of the different studies ranges from 31 to 68%. Even though the underlying calculations might be different, this may suggest selection bias. This is further suggested by some of the sample characteristics, e.g. 63% of the Portuguese participants were female. This might mean that the study populations may differ in terms of socio-demographic characteristics, and possibly also physical activity and sedentary time levels, which might have influenced the results and hampers the comparability across studies. Weighting data towards the population distribution for key characteristics such as age, sex and educational level would partly solve this problem. However, the majority of the included studies did not have such a weighting system. More importantly, such a system would still not adjust for selection bias caused by fewer or more sedentary/active individuals agreeing to take part in the study. Guidelines on how to deal with such differences between study samples in population-based studies would be useful in international population surveillance, especially when the data are pooled.

In addition, differences between studies with regard to the data collection could have influenced the results and might partly explain the observed differences between countries in the current analysis. For example, the Swedish ABC study was conducted in 2001–2002, while the other data were collected between 2006 and 2009. In addition, the Portuguese participants were asked to wear the accelerometer for at least 4 days, including at least 2 weekend days, but were encouraged to wear it longer. The other studies all asked their participants to wear the accelerometer for at least 7 days. These different strategies have resulted in a different number of valid days (with Portugal having approximately 2 days less) and might have led to an over-representation of weekend days in the Portuguese data.

Moreover, we found that several socio-demographic characteristics were difficult to harmonise across studies, which is illustrated by the crude harmonisation of the educational level of the participants, and the inability to harmonise any other characteristics in addition to sex, age, weight status and education. In addition, since some of the studies used 60 s epochs in their data collection, we were forced to apply this setting to all data files. This meant that we lost detail of the data at the expense of comparability. Finally, a known limitation of accelerometers is their inability to capture certain movements, especially those without a strong vertical component, such as cycling. Since we also know there are cultural differences in activity behaviours across countries/regions in Europe, this might mean that some of the observed differences might be partly explained by these limitations of the accelerometers.

Overall, while the pooling of existing accelerometer data has substantial advantages over comparing self-reported data from different countries, there are still some limitations. These could be overcome by further standardisation in data collection and interpretation across countries or by installing a cross-European surveillance system, using the same standardised protocol across countries. A recently published expert consensus provides an overview of the strategies and utilities needed to enable cross-country comparison of accelerometer data, both for historical and future data collection [11].

### Future Recommendations

The results of this study demonstrate the utility of objective measures in sedentary behaviour and physical inactivity population surveillance and research. Setting up a cross-European accelerometer-based surveillance system using standardised protocols for data collection in all involved countries simultaneously would enable more insight into the prevalence, diversity and correlates of sedentary behaviour and physical inactivity across Europe. Assessing a wide variety of possible correlates of sedentary behaviour and physical inactivity would provide a better notion of the individuals and groups at risk, which could then be targeted in public health strategies, as well as the opportunity to investigate the differences across countries in-depth. The major challenge for accelerometer-based surveillance is budget, as using accelerometers is more costly than using questionnaires. In particular, cross-country efforts are challenging to fund (and organise); however, they have clear additional benefits over national surveillance when it comes to comparability of the data.

## Conclusions

This study shows high levels of sedentary time and physical inactivity across European adults. Participants from Norway were the most sedentary, whereas participants from England were the most inactive. In general, interventions and policies aiming to improve these lifestyle behaviours should pay special attention to older people and people with overweight or obesity, who are more likely to be sedentary and physically inactive. Additionally, sex and socio-economic differences should be taken into account as men and highly educated people tend to be more active but also more sedentary than women and people with lower levels of education, respectively. As people who are classified as high sedentary/low active might be most at risk of the associated non-communicable diseases, this combination of behaviours deserves specific attention in both research as well as in intervention and policy actions. It is recommended that these behaviours be monitored across Europe using an accelerometer-based surveillance system.

## Electronic supplementary material

Below is the link to the electronic supplementary material.

Supplementary material 1 (DOCX 86 kb)
