# Supplementary material for: Sedentary Time and Physical Activity Surveillance Through Accelerometer Pooling in Four European Countries
Source: Sports Med. 2016 Dec 10;47(7):1421–35. doi: 10.1007/s40279-016-0658-y (PMC5488150; doi:10.1007/s40279-016-0658-y)
Supplement: Supplementary file 1 — Supplementary material 1 (DOCX 86 kb) [file 40279_2016_658_MOESM1_ESM.docx]

Table S1. Accelerometer assessed sedentary time and physical activity in the total sample and the separate study samples – per subgroup based on gender, age, BMI and educational level.

| **MEN** | **Total** | **England** | **Norway** | **Portugal** | **Sweden** | |
| --- | --- | --- | --- | --- | --- | --- |
|  |  |  |  |  | *ABC study* | *SNAP study* |
| Mean (SD) number of valid days  Mean (SD) min/day wear time  Mean (SD) activity kcounts/day  Mean (SD) min/day sed time  Mean (SD) min/day light PA  Mean (SD) min/day moderate PA  Mean (SD) min/day vigorous PA  Mean (SD) min/day MVPA  Percentage (SD) sed of wear time  Percentage (SD) light PA of wear time  Percentage (SD) moderate PA of wear time  Percentage (SD) vigorous PA of wear time  Percentage (SD) MVPA of wear time  Median (IQR) number ≥30 min sed bouts  Mean (SD) min/day ≥30 min sed bouts  Median (IQR) number ≥60 min sed bouts  Mean (SD) min/day ≥60 min sed bouts  Median (IQR) number ≥10 min MVPA bouts^a^  Mean (SD) min/day ≥10 min MVPA bouts^a^  Percentage more than 7.5 h/day sed  Percentage more than 10 h/day sed  Percentage not meeting PA recs - based on total time in MVPA  Percentage not meeting PA recs - based on time in ≥10 min MVPA bouts^a^  Percentage more than 10 h/day sed AND not meeting PA recs based on total MVPA | 6.47 (1.15)  877.20 (74.68)  314.86 (134.66)  548.29 (93.83)  290.48 (84.84)  35.63 (22.82)  2.81 (6.97)  38.44 (25.27)  62.56% (9.66)  33.06% (9.09)  4.06% (2.60)  0.32% (0.80)  4.38% (2.87)  3.13 (2.00-4.20)  146.70 (76.93)  0.33 (0.14-0.63)  35.45 (37.29)  0.57 (0.17-1.17)  16.19 (18.88)  85.2%  31.0%  27.6%  72.3%  11.0% | 6.05 (0.82)  859.84 (74.46)  296.45 (145.91)  536.02 (98.99)  288.71 (87.45)  33.37 (25.96)  1.73 (5.09)  35.1 (27.48)  62.42% (10.74)  33.52% (9.64)  3.86% (2.93)  0.20% (0.58)  4.06% (3.11)  3.00 (1.86-4.33)  145.72 (82.02)  0.29 (0.14-0.57)  32.79 (35.67)  0.50 (0.14-1.14)  14.02 (19.03)  81.0%  27.4%  37.4%  77.7%  15.1% | 6.86 (0.95)  897.08 (68.64)  310.36 (129.03)  569.32 (83.66)  290.92 (79.25)  34.21 (21.85)  2.63 (6.75)  36.84 (24.31)  63.56% (8.64)  32.34% (8.15)  3.81% (2.43)  0.29% (0.74)  4.10% (2.68)  3.29 (2.29-4.43)  154.91 (74.91)  0.40 (0.14-0.71)  37.95 (35.63)  0.57 (0.14-1.14)  16.92 (19.83)  91.6%  37.7%  28.5%  71.7%  13.5% | 4.68 (1.05)  837.16 (74.24)  312.10 (133.93)  503.68 (91.37)  295.93 (87.88)  34.94 (22.90)  2.61 (7.01)  37.55 (25.38)  60.27% (10.14)  35.25% (9.73)  4.18% (2.77)  0.31% (0.85)  4.49% (3.05)  2.85 (2.00-3.91)  138.39 (72.23)  0.33 (0.16-0.63)  35.68 (39.75)  0.50 (0.00-1.00)  14.83 (19.17)  74.7%  15.4%  28.3%  76.5%  6.1% | 6.83 (1.21)  881.06 (73.26)  326.80 (137.49)  507.88 (98.96)  337.20 (92.94)  33.65 (22.56)  2.34 (5.34)  35.99 (24.49)  57.72% (10.46)  38.20% (9.66)  3.82% (2.51)  0.27% (0.61)  4.08% (2.74)  2.43 (1.57-3.52)  121.58 (76.00)  0.29 (0.14-0.50)  31.02 (44.86)  0.43 (0.14-0.86)  11.92 (14.27)  71.9%  18.3%  31.9%  80.2%  8.3% | 6.51 (0.90)  878.60 (73.96)  331.07 (129.98)  565.67 (87.73)  268.27 (76.13)  40.47 (20.87)  4.18 (8.73)  44.65 (24.02)  64.41% (8.60)  30.48% (8.12)  4.63% (2.41)  0.48% (1.01)  5.11% (2.79)  3.14 (2.23-4.33)  150.91 (75.28)  0.33 (0.14-0.67)  35.98 (35.69)  0.80 (0.33-1.50)  19.32 (18.53)  90.1%  36.9%  16.6%  63.8%  7.6% |
| **WOMEN** | **Total** | **England** | **Norway** | **Portugal** | **Sweden** | |
|  |  |  |  |  | *ABC study* | *SNAP study* |
| Mean (SD) number of valid days  Mean (SD) min/day wear time  Mean (SD) activity kcounts/day  Mean (SD) min/day sed time  Mean (SD) min/day light PA  Mean (SD) min/day moderate PA  Mean (SD) min/day vigorous PA  Mean (SD) min/day MVPA  Percentage (SD) sed of wear time  Percentage (SD) light PA of wear time  Percentage (SD) moderate PA of wear time  Percentage (SD) vigorous PA of wear time  Percentage (SD) MVPA of wear time  Median (IQR) number ≥30 min sed bouts  Mean (SD) min/day ≥30 min sed bouts  Median (IQR) number ≥60 min sed bouts  Mean (SD) min/day ≥60 min sed bouts  Median (IQR) number ≥10 min MVPA bouts^a^  Mean (SD) min/day ≥10 min MVPA bouts^a^  Percentage more than 7.5 h/day sed  Percentage more than 10 h/day sed  Percentage not meeting PA recs - based on total time in MVPA  Percentage not meeting PA recs - based on time in ≥10 min MVPA bouts^a^  Percentage more than 10 h/day sed AND not meeting PA recs based on total MVPA | 6.40 (1.15)  863.45 (70.34)  298.16 (120.69)  515.55 (87.61)  314.53 (80.20)  31.75 (21.96)  1.62 (4.52)  33.37 (23.36)  59.74% (9.15)  36.41% (8.77)  3.66% (2.47)  0.19% (0.52)  3.85% (2.63)  2.57 (1.71-3.50)  121.89 (66.90)  0.29 (0.13-0.50)  27.73 (30.96)  0.57 (0.14-1.17)  15.67 (17.59)  77.8%  16.2%  35.3%  71.9%  6.9% | 6.40 (0.91)  840.50 (68.55)  259.49 (111.04)  505.78 (82.64)  309.60 (78.94)  24.34 (19.46)  0.79 (3.03)  25.13 (20.38)  60.27% (9.18)  36.74% (8.49)  2.89% (2.30)  0.09% (0.35)  2.99% (2.40)  2.57 (1.6-3.43)  117.84 (66.00)  0.2 (0.00-0.43)  24.46 (28.25)  0.29 (0.00-0.86)  9.96 (14.36)  75.2%  11.7%  52.7%  84.3%  8.2% | 6.87 (0.87)  886.19 (64.29)  302.18 (118.69)  538.79 (79.85)  313.55 (77.49)  31.72 (20.73)  2.14 (5.30)  33.85 (22.57)  60.88% (8.42)  35.31% (8.07)  3.57% (2.33)  0.24% (0.60)  3.81% (2.54)  2.60 (1.75-3.57)  126.26 (66.39)  0.29 (0.14-0.57)  29.76 (31.61)  0.67 (0.25-1.25)  18.54 (18.74)  86.1%  21.8%  33.3%  65.5%  9.4% | 4.83 (1.24)  840.91 (76.78)  293.21 (125.83)  474.58 (98.52)  336.09 (90.14)  29.42 (22.12)  0.82 (3.45)  30.24 (22.96)  56.48% (10.74)  39.96% (10.08)  3.46% (2.55)  0.09% (0.41)  3.56% (2.64)  2.25 (1.50-3.25)  110.40 (66.09)  0.25 (0.00-0.50)  26.29 (30.96)  0.4 (0.00-0.93)  11.64 (15.13)  61.9%  10.1%  43.2%  81.1%  4.7% | 6.81 (0.98)  863.76 (67.44)  304.20 (113.45)  489.56 (85.20)  343.88 (82.53)  29.12 (19.78)  1.20 (3.32)  30.32 (20.81)  56.74% (9.17)  39.76% (8.80)  3.36% (2.22)  0.14% (0.38)  3.50% (2.34)  2.33 (1.50-3.29)  116.24 (69.38)  0.29 (0.14-0.50)  30.28 (37.44)  0.43 (0.14-0.86)  12.24 (14.66)  67.6%  10.5%  39.7%  78.6%  6.1% | 6.49 (0.86)  864.45 (66.96)  322.05 (123.25)  528.34 (82.69)  294.03 (70.18)  39.83 (23.47)  2.24 (5.15)  42.08 (24.82)  61.09% (8.21)  34.05% (7.87)  4.60% (2.53)  0.26% (0.59)  4.86% (2.70)  2.75 (1.86-3.80)  128.42 (66.38)  0.29 (0.14-0.50)  27.30 (28.50)  0.86 (0.33-1.57)  20.11 (18.82)  82.7%  18.5%  18.2%  62.6%  4.4% |

| **20-35 YEARS** | **Total** | **England** | **Norway** | **Portugal** | **Sweden** | |
| --- | --- | --- | --- | --- | --- | --- |
|  |  |  |  |  | *ABC study* | *SNAP study* |
| Mean (SD) number of valid days  Mean (SD) min/day wear time  Mean (SD) activity kcounts/day  Mean (SD) min/day sed time  Mean (SD) min/day light PA  Mean (SD) min/day moderate PA  Mean (SD) min/day vigorous PA  Mean (SD) min/day MVPA  Percentage (SD) sed of wear time  Percentage (SD) light PA of wear time  Percentage (SD) moderate PA of wear time  Percentage (SD) vigorous PA of wear time  Percentage (SD) MVPA of wear time  Median (IQR) number ≥30 min sed bouts  Mean (SD) min/day ≥30 min sed bouts  Median (IQR) number ≥60 min sed bouts  Mean (SD) min/day ≥60 min sed bouts  Median (IQR) number ≥10 min MVPA bouts^a^  Mean (SD) min/day ≥10 min MVPA bouts^a^  Percentage more than 7.5 h/day sed  Percentage more than 10 h/day sed  Percentage not meeting PA recs - based on total time in MVPA  Percentage not meeting PA recs - based on time in ≥10 min MVPA bouts^a^  Percentage more than 10 h/day sed AND not meeting PA recs based on total MVPA | 6.21 (1.26)  860.76 (70.08)  319.87 (123.70)  524.93 (92.50)  297.01 (86.42)  35.51 (21.11)  3.30 (6.84)  38.81 (23.78)  61.01% (9.62)  34.48% (9.48)  4.13% (2.45)  0.38% (0.81)  4.52% (2.77)  2.50 (1.57-3.67)  123.28 (72.06)  0.25 (0.00-0.50)  29.42 (35.19)  0.63 (0.20-1.29)  16.22 (17.83)  78.9%  21.7%  25.3%  72.1%  6.0% | 6.18 (0.97)  836.99 (69.85)  299.43 (125.86)  509.07 (96.95)  293.25 (79.89)  32.30 (22.01)  2.38 (5.68)  34.67 (23.89)  60.75% (9.91)  35.12% (9.34)  3.86% (2.57)  0.28% (0.65)  4.14% (2.78)  2.29 (1.40-3.50)  112.57 (70.07)  0.20 (0.00-0.46)  23.72 (27.68)  0.50 (0.14-1.16)  13.94 (17.39)  72.9%  17.1%  35.2%  76.4%  6.2% | 6.73 (1.06)  883.17 (64.87)  309.11 (115.70)  547.99 (84.39)  298.99 (83.96)  32.92 (19.40)  3.27 (6.23)  36.19 (21.99)  62.13% (8.95)  33.76% (8.84)  3.73% (2.22)  0.37% (0.72)  4.11% (2.52)  2.57 (1.59-3.71)  126.17 (72.60)  0.29 (0.13-0.57)  31.92 (36.12)  0.63 (0.25-1.21)  16.60 (17.54)  87.4%  27.7%  28.8%  71.4%  9.9% | 4.64 (1.11)  844.89 (71.56)  310.69 (116.65)  500.13 (87.21)  309.67 (88.66)  32.42 (20.19)  2.67 (6.51)  35.10 (22.43)  59.30% (9.56)  36.54% (9.45)  3.85% (2.43)  0.32% (0.77)  4.16% (2.69)  2.50 (1.75-3.50)  121.46 (63.08)  0.25 (0.00-0.5)  27.84 (30.92)  0.50 (0.00-1.00)  2.69 (16.22)  73.3%  13.2%  30.8%  79.9%  3.5% | 6.67 (1.23)  866.53 (73.92)  340.64 (128.99)  495.90 (96.06)  331.85 (91.83)  35.80 (21.70)  2.98 (5.86)  38.78 (23.82)  57.30% (10.10)  38.25% (9.79)  4.11% (2.36)  0.35% (0.69)  4.45% (2.63)  2.17 (1.31-3.17)  109.28 (73.39)  0.20 (0.00-0.50)  28.14 (46.11)  0.50 (0.17-1.00)  13.46 (15.27)  68.4%  13.8%  22.6%  76.8%  4.0% | 6.30 (0.94)  855.43 (64.35)  342.98 (128.66)  539.60 (89.98)  267.98 (79.79)  43.22 (20.96)  4.63 (8.73)  47.85 (24.53)  63.04% (9.20)  31.34% (9.08)  5.07% (2.47)  0.55% (1.05)  5.62% (2.91)  2.86 (1.86-4.00)  137.07 (74.84)  0.29 (0.14-0.60)  32.15 (33.41)  1.00 (0.43-1.71)  21.35 (19.72)  81.9%  27.2%  11.4%  62.0%  3.3% |

| **36-50 YEARS** | **Total** | **England** | **Norway** | **Portugal** | **Sweden** | |
| --- | --- | --- | --- | --- | --- | --- |
|  |  |  |  |  | *ABC study* | *SNAP study* |
| Mean (SD) number of valid days  Mean (SD) min/day wear time  Mean (SD) activity kcounts/day  Mean (SD) min/day sed time  Mean (SD) min/day light PA  Mean (SD) min/day moderate PA  Mean (SD) min/day vigorous PA  Mean (SD) min/day MVPA  Percentage (SD) sed of wear time  Percentage (SD) light PA of wear time  Percentage (SD) moderate PA of wear time  Percentage (SD) vigorous PA of wear time  Percentage (SD) MVPA of wear time  Median (IQR) number ≥30 min sed bouts  Mean (SD) min/day ≥30 min sed bouts  Median (IQR) number ≥60 min sed bouts  Mean (SD) min/day ≥60 min sed bouts  Median (IQR) number ≥10 min MVPA bouts^a^  Mean (SD) min/day ≥10 min MVPA bouts^a^  Percentage more than 7.5 h/day sed  Percentage more than 10 h/day sed  Percentage not meeting PA recs - based on total time in MVPA  Percentage not meeting PA recs - based on time in ≥10 min MVPA bouts^a^  Percentage more than 10 h/day sed AND not meeting PA recs based on total MVPA | 6.48 (1.12)  880.23 (70.01)  323.16 (120.75)  527.07 (92.55)  315.67 (80.66)  34.97 (20.55)  2.51 (5.85)  37.49 (22.62)  59.85% (9.24)  35.90% (8.88)  3.97% (2.33)  0.28% (0.65)  4.26% (2.55)  2.56 (1.70-3.57)  121.87 (67.15)  0.29 (0.13-0.50)  26.89 (29.83)  0.57 (0.17-1.14)  15.75 (16.62)  79.7%  22.2%  26.0%  71.9%  6.7% | 6.35 (0.92)  851.64 (72.73)  314.87 (121.41)  494.64 (89.56)  321.76 (82.81)  33.73 (22.96)  1.52 (3.94)  35.25 (24.19)  58.11% (9.51)  37.76% (9.07)  3.95% (2.65)  0.18% (0.46)  4.13% (2.80)  2.25 (1.40-3.18)  107.45 (65.57)  0.17 (0.00-0.43)  22.23 (28.11)  0.50 (0.14-1.14)  13.41 (15.99)  69.0%  13.3%  33.1%  77.5%  5.2% | 6.89 (0.91)  902.38 (61.91)  314.71 (116.82)  554.00 (80.84)  313.27 (73.88)  32.37 (18.91)  2.73 (5.94)  35.11 (21.47)  61.42% (8.1)  34.70% (7.76)  3.58% (2.07)  0.30% (0.64)  3.88% (2.33)  2.71 (1.86-3.71)  128.42 (64.09)  0.29 (0.14-0.57)  29.08 (28.42)  0.57 (0.17-1.00)  16.42 (16.75)  88.8%  29.4%  28.7%  70.9%  10.4% | 5.06 (1.45)  855.62 (70.54)  330.16 (137.66)  480.21 (104.29)  337.95 (92.28)  35.69 (23.15)  1.78 (6.01)  37.47 (25.05)  56.11% (11.23)  39.54% (10.56)  4.14% (2.62)  0.21% (0.74)  4.35% (2.84)  2.17 (1.31-3.25)  108.58 (68.88)  0.25 (0.00-0.50)  24.86 (29.44)  0.43 (0.00-1.00)  14.11 (18.29)  64.8%  12.3%  29.2%  76.2%  5.7% | 6.79 (1.08)  883.55 (70.57)  331.78 (126.90)  487.90 (97.08)  362.12 (87.20)  31.84 (20.58)  1.69 (3.94)  33.53 (21.92)  55.22% (9.94)  40.99% (9.24)  3.60% (2.29)  0.19% (0.44)  3.79% (2.44)  2.14 (1.35-3.00)  104.95 (67.31)  0.17 (0.00-0.43)  24.70 (38.34)  0.43 (0.14-0.85)  10.99 (12.37)  61.6%  14.3%  34.5%  82.3%  7.9% | 6.48 (0.88)  877.00 (68.49)  331.48 (115.55)  542.67 (84.82)  291.67 (70.39)  39.36 (19.32)  3.30 (6.83)  42.66 (21.54)  61.83% (8.13)  33.30% (7.82)  4.50% (2.21)  0.37% (0.76)  4.87% (2.46)  2.86 (2.00-3.86)  132.15 (67.67)  0.29 (0.14-0.50)  28.21 (28.71)  0.83 (0.33-1.43)  18.37 (16.90)  86.3%  24.5%  15.4%  65.1%  3.3% |

| **51-66 YEARS** | **Total** | **England** | **Norway** | **Portugal** | **Sweden** | |
| --- | --- | --- | --- | --- | --- | --- |
|  |  |  |  |  | *ABC study* | *SNAP study* |
| Mean (SD) number of valid days  Mean (SD) min/day wear time  Mean (SD) activity kcounts/day  Mean (SD) min/day sed time  Mean (SD) min/day light PA  Mean (SD) min/day moderate PA  Mean (SD) min/day vigorous PA  Mean (SD) min/day MVPA  Percentage (SD) sed of wear time  Percentage (SD) light PA of wear time  Percentage (SD) moderate PA of wear time  Percentage (SD) vigorous PA of wear time  Percentage (SD) MVPA of wear time  Median (IQR) number ≥30 min sed bouts  Mean (SD) min/day ≥30 min sed bouts  Median (IQR) number ≥60 min sed bouts  Mean (SD) min/day ≥60 min sed bouts  Median (IQR) number ≥10 min MVPA bouts^a^  Mean (SD) min/day ≥10 min MVPA bouts^a^  Percentage more than 7.5 h/day sed  Percentage more than 10 h/day sed  Percentage not meeting PA recs - based on total time in MVPA  Percentage not meeting PA recs - based on time in ≥10 min MVPA bouts^a^  Percentage more than 10 h/day sed AND not meeting PA recs based on total MVPA | 6.60 (1.03)  874.79 (72.65)  299.08 (129.95)  537.41 (90.19)  302.27 (81.05)  33.62 (24.26)  1.48 (5.06)  35.10 (25.65)  61.48% (9.26)  34.53% (8.73)  3.83% (2.67)  0.17% (0.57)  3.99% (2.83)  3.00 (2.00-4.00)  141.10 (72.11)  0.29 (0.14-0.57)  33.41 (34.43)  0.57 (0.14-1.29)  16.88 (19.61)  83.8%  24.5%  34.7%  69.8%  10.4% | 6.61 (0.75)  860.70 (70.47)  271.75 (131.38)  530.07 (88.27)  301.90 (83.98)  27.96 (24.26)  0.77 (3.41)  28.73 (25.02)  61.73% (9.82)  34.97% (8.92)  3.22% (2.72)  0.09% (0.38)  3.31% (2.81)  3.00 (2.00-4.00)  139.16 (73.00)  0.29 (0.14-0.57)  29.84 (33.76)  0.33 (0.00-0.86)  11.68 (18.07)  81.5%  20.9%  48.5%  82.1%  13.6 | 6.93 (0.81)  894.98 (66.01)  307.31 (130.40)  555.52 (84.07)  303.64 (78.73)  34.10 (23.50)  1.72 (5.70)  35.83 (25.37)  62.15% (8.70)  33.87% (8.18)  3.80% (2.59)  0.19% (0.64)  3.99% (2.80)  3.14 (2.25-4.29)  148.83 (71.48)  0.33 (0.14-0.67)  36.18 (34.51)  0.63 (0.17-1.29)  19.42 (21.42)  89.3%  29.9%  32.1%  64.5%  11.7% | 4.70 (1.06)  841.83 (80.66)  305.56 (124.24)  476.91 (98.69)  330.20 (84.75)  33.83 (23.67)  0.90 (3.56)  34.72 (24.63)  56.60% (10.10)  39.29% (9.71)  4.01% (2.79)  0.11% (0.42)  4.12% (2.90)  2.25 (1.50-3.25)  114.71 (66.86)  0.25 (0.00-0.50)  29.02 (31.17)  0.50 (0.14-1.00)  14.72 (17.25)  63.1%  10.3%  38.4%  75.7%  3.4% | 6.94 (1.00)  869.77 (67.85)  287.13 (108.67)  505.73 (83.28)  335.59 (82.03)  27.64 (19.03)  0.82 (2.84)  28.46 (19.92)  58.24% (9.03)  38.49% (8.51)  3.18% (2.18)  0.09% (0.32)  3.27% (2.28)  2.67 (1.83-3.57)  127.95 (68.51)  0.29 (0.14-0.57)  34.05 (36.46)  0.33 (0.00-0.86)  11.12 (13.12)  76.1%  13.3%  44.8%  80.5%  7.1% | 6.62 (0.82)  872.52 (74.51)  311.47 (134.19)  550.45 (87.35)  280.72 (73.17)  39.24 (25.63)  2.11 (6.05)  41.35 (26.94)  63.11% (8.57)  32.17% (7.93)  4.48% (2.70)  0.24% (0.70)  4.73% (2.87)  3.00 (2.14-4.14)  145.82 (72.61)  0.33 (0.14-0.57)  33.76 (34.66)  0.86 (0.33-1.50)  20.35 (19.79)  87.8%  28.8%  22.9%  61.7%  9.9% |

| **67-75 YEARS** | **Total** | **England** | **Norway** | **Portugal** | **Sweden** | |
| --- | --- | --- | --- | --- | --- | --- |
|  |  |  |  |  | *ABC study* | *SNAP study* |
| Mean (SD) number of valid days  Mean (SD) min/day wear time  Mean (SD) activity kcounts/day  Mean (SD) min/day sed time  Mean (SD) min/day light PA  Mean (SD) min/day moderate PA  Mean (SD) min/day vigorous PA  Mean (SD) min/day MVPA  Percentage (SD) sed of wear time  Percentage (SD) light PA of wear time  Percentage (SD) moderate PA of wear time  Percentage (SD) vigorous PA of wear time  Percentage (SD) MVPA of wear time  Median (IQR) number ≥30 min sed bouts  Mean (SD) min/day ≥30 min sed bouts  Median (IQR) number ≥60 min sed bouts  Mean (SD) min/day ≥60 min sed bouts  Median (IQR) number ≥10 min MVPA bouts^a^  Mean (SD) min/day ≥10 min MVPA bouts^a^  Percentage more than 7.5 h/day sed  Percentage more than 10 h/day sed  Percentage not meeting PA recs - based on total time in MVPA  Percentage not meeting PA recs - based on time in ≥10 min MVPA bouts^a^  Percentage more than 10 h/day sed AND not meeting PA recs based on total MVPA | 6.15 (1.27)  835.75 (74.48)  236.83 (123.31)  527.14 (92.56)  284.60 (85.50)  23.35 (22.19)  0.67 (4.45)  24.02 (23.25)  63.17% (10.22)  33.99% (9.59)  2.77% (2.62)  0.08% (0.51)  2.85% (2.73)  3.29 (2.40-4.57)  163.89 (80.31)  0.43 (0.17-0.75)  41.80 (40.79)  0.29 (0.00-0.86)  12.31 (18.43)  81.2%  21.8%  56.6%  80.6%  15.8% | 6.58 (0.78)  835.69 (72.11)  194.65 (101.77)  550.21 (83.10)  269.04 (77.80)  16.17 (16.46)  0.27 (3.09)  16.44 (17.77)  65.96% (9.19)  32.09% (8.59)  1.92% (1.94)  0.03% (0.35)  1.95% (2.08)  3.57 (2.57-4.71)  171.82 (79.19)  0.43 (0.14-0.71)  40.30 (35.92)  0.14 (0.00-0.57)  6.83 (12.83)  90.1%  25.5%  73.1%  91.5%  22.8% | 6.88 (0.88)  853.98 (73.83)  261.10 (131.42)  551.27 (84.16)  271.32 (79.41)  30.07 (24.70)  1.33 (6.63)  31.40 (25.83)  64.69% (9.08)  31.66% (8.38)  3.50% (2.88)  0.15% (0.75)  3.65% (3.01)  3.75 (2.67-4.75)  177.82 (81.02)  0.43 (0.20-0.83)  44.48 (40.32)  0.57 (0.14-1.25)  19.55 (22.59)  88.9%  28.9%  41.5%  66.6%  16.0% | 4.65 (0.87)  811.11 (74.85)  246.09 (121.42)  482.95 (95.20)  304.92 (95.32)  22.92 (21.21)  0.31 (2.53)  23.23 (21.84)  59.70% (11.30)  37.45% (10.78)  2.81% (2.61)  0.04% (0.31)  2.85% (2.69)  3.00 (2.00-3.80)  141.24 (76.52)  0.33 (0.00-0.75)  38.93 (45.40)  0.25 (0.00-0.75)  9.57 (14.65)  64.8%  12.2%  55.2%  86.3%  8.5% | 6.92 (0.81)  850.92 (62.85)  267.74 (131.38)  508.40 (87.87)  315.06 (77.58)  26.53 (25.49)  0.92 (3.89)  27.46 (26.36)  59.77% (9.58)  37.04% (8.75)  3.09% (2.89)  0.10% (0.43)  3.19% (2.97)  3.14 (2.29-4.20)  161.62 (79.39)  0.43 (0.14-0.86)  46.49 (42.23)  0.33 (0.00-1.00)  15.15 (21.25)  76.8%  15.8%  54.7%  72.6%  13.7% |  |

| **BMI < 25** | **Total** | **England** | **Norway** | **Portugal** | **Sweden** | |
| --- | --- | --- | --- | --- | --- | --- |
|  |  |  |  |  | *ABC study* | *SNAP study* |
| Mean (SD) number of valid days  Mean (SD) min/day wear time  Mean (SD) activity kcounts/day  Mean (SD) min/day sed time  Mean (SD) min/day light PA  Mean (SD) min/day moderate PA  Mean (SD) min/day vigorous PA  Mean (SD) min/day MVPA  Percentage (SD) sed of wear time  Percentage (SD) light PA of wear time  Percentage (SD) moderate PA of wear time  Percentage (SD) vigorous PA of wear time  Percentage (SD) MVPA of wear time  Median (IQR) number ≥30 min sed bouts  Mean (SD) min/day ≥30 min sed bouts  Median (IQR) number ≥60 min sed bouts  Mean (SD) min/day ≥60 min sed bouts  Median (IQR) number ≥10 min MVPA bouts^a^  Mean (SD) min/day ≥10 min MVPA bouts^a^  Percentage more than 7.5 h/day sed  Percentage more than 10 h/day sed  Percentage not meeting PA recs - based on total time in MVPA  Percentage not meeting PA recs - based on time in ≥10 min MVPA bouts^a^  Percentage more than 10 h/day sed AND not meeting PA recs based on total MVPA | 6.47 (1.13)  871.83 (69.80)  327.84 (132.47)  527.78 (90.30)  304.00 (83.32)  37.00 (23.09)  3.05 (6.84)  40.05 (25.41)  60.57% (9.36)  34.85% (9.06)  4.24% (2.58)  0.35% (0.78)  4.59% (2.85)  2.71 (1.71-3.71)  128.89 (71.74)  0.29 (0.14-0.57)  30.19 (34.40)  0.71 (0.25-1.43)  18.82 (19.76)  80.4%  22.1%  24.1%  65.6%  6.3% | 6.39 (0.91)  845.99 (69.68)  302.27 (145.47)  507.15 (92.54)  304.12 (85.64)  32.73 (26.05)  1.99 (5.49)  34.72 (28.08)  60.02% (10.19)  35.90% (9.48)  3.85% (3.00)  0.23% (0.63)  4.08% (3.23)  2.43 (1.43-3.57)  117.02 (70.32)  0.17 (0.00-0.45)  24.34 (27.14)  0.50 (0.14-1.20)  15.62 (21.59)  73.0%  16.1%  37.7%  74.7%  7.7% | 6.86 (0.90)  890.08 (64.57)  324.86 (132.36)  546.77 (82.27)  304.41 (80.54)  35.56 (22.29)  3.34 (7.51)  38.89 (25.10)  61.51% (8.70)  34.12% (8.38)  3.99% (2.50)  0.37% (0.84)  4.36% (2.80)  2.71 (1.83-3.83)  133.00 (71.41)  0.29 (0.14-0.57)  32.24 (34.31)  0.71 (0.29-1.29)  20.60 (21.20)  87.4%  26.7%  25.4%  62.2%  8.5% | 4.67 (1.07)  842.16 (71.93)  311.89 (128.88)  488.18 (93.23)  318.94 (91.59)  33.14 (22.57)  1.90 (5.37)  35.04 (24.34)  58.06% (10.42)  37.79% (10.06)  3.93% (2.64)  0.22% (0.64)  4.15% (2.85)  2.33 (1.60-3.5)  118.13 (66.23)  0.25 (0.00-0.50)  27.00 (29.82)  0.50 (0.00-1.00)  13.43 (17.51)  67.4%  12.2%  32.6%  78.1%  4.5% | 6.80 (1.16)  872.16 (68.91)  334.40 (128.57)  494.66 (96.01)  340.81 (89.13)  34.38 (21.07)  2.31 (5.15)  36.69 (22.96)  56.75% (10.07)  39.06% (9.59)  3.93% (2.34)  0.26% (0.59)  4.20% (2.57)  2.27 (1.43-3.38)  115.63 (75.83)  0.29 (0.14-0.50)  30.22 (45.27)  0.57 (0.14-1.09)  13.96 (15.15)  66.8%  14.5%  28.4%  74.5%  6.3% | 6.51 (0.85)  870.96 (68.71)  342.67 (128.21)  540.35 (85.47)  284.54 (74.09)  42.29 (22.76)  3.78 (7.38)  46.07 (24.80)  62.03% (8.48)  32.67% (8.17)  4.86% (2.47)  0.44% (0.86)  5.30% (2.74)  2.86 (2.00-3.86)  137.37 (70.96)  0.29 (0.14-0.57)  31.11 (32.96)  1.00 (0.43-1.60)  21.67 (18.90)  85.0%  25.3%  13.5%  58.5%  4.1% |

| **BMI 25 – 30** | **Total** | **England** | **Norway** | **Portugal** | **Sweden** | |
| --- | --- | --- | --- | --- | --- | --- |
|  |  |  |  |  | *ABC study* | *SNAP study* |
| Mean (SD) number of valid days  Mean (SD) min/day wear time  Mean (SD) activity kcounts/day  Mean (SD) min/day sed time  Mean (SD) min/day light PA  Mean (SD) min/day moderate PA  Mean (SD) min/day vigorous PA  Mean (SD) min/day MVPA  Percentage (SD) sed of wear time  Percentage (SD) light PA of wear time  Percentage (SD) moderate PA of wear time  Percentage (SD) vigorous PA of wear time  Percentage (SD) MVPA of wear time  Median (IQR) number ≥30 min sed bouts  Mean (SD) min/day ≥30 min sed bouts  Median (IQR) number ≥60 min sed bouts  Mean (SD) min/day ≥60 min sed bouts  Median (IQR) number ≥10 min MVPA bouts^a^  Mean (SD) min/day ≥10 min MVPA bouts^a^  Percentage more than 7.5 h/day sed  Percentage more than 10 h/day sed  Percentage not meeting PA recs - based on total time in MVPA  Percentage not meeting PA recs - based on time in ≥10 min MVPA bouts^a^  Percentage more than 10 h/day sed AND not meeting PA recs based on total MVPA | 6.44 (1.17)  871.57 (73.21)  299.12 (119.30)  531.14 (92.28)  306.22 (82.70)  32.61 (21.53)  1.60 (4.76)  34.21 (22.89)  60.96% (9.44)  35.13% (9.01)  3.73% (2.44)  0.18% (0.55)  3.91% (2.59)  2.75 (1.86-3.86)  134.00 (71.84)  0.29 (0.14-0.57)  31.65 (32.99)  0.57 (0.14-1.03)  14.73 (16.73)  81.8%  23.5%  33.1%  74.8%  9.6% | 6.53 (0.78)  853.51 (72.07)  286.17 (119.19)  516.76 (87.83)  305.47 (80.92)  30.15 (22.47)  1.11 (3.35)  31.27 (23.20)  60.61% (9.41)  35.75% (8.81)  3.51% (2.54)  0.13% (0.38)  3.64% (2.63)  2.71 (1.8-3.79)  128.27 (71.42)  0.29 (0.14-0.50)  27.79 (30.64)  0.43 (0.14-1.00)  12.16 (14.94)  78.0%  17.9%  41.4%  80.0%  9.9% | 6.89 (0.93)  892.70 (65.66)  295.40 (112.67)  556.94 (82.38)  302.61 (78.02)  31.61 (20.27)  1.55 (3.99)  33.16 (21.49)  62.46% (8.53)  33.84% (8.15)  3.53% (2.24)  0.17% (0.45)  3.70% (2.37)  3.00 (2.14-4.00)  145.23 (71.97)  0.33 (0.14-0.63)  35.81 (33.56)  0.57 (0.17-1.00)  16.10 (17.28)  89.8%  31.5%  33.7%  72.0%  12.9% | 4.77 (1.24)  843.96 (76.46)  307.21 (130.94)  482.10 (102.70)  327.99 (94.12)  32.34 (21.83)  1.53 (5.60)  33.87 (23.53)  57.16% (11.23)  38.85% (10.60)  3.82% (2.56)  0.18% (0.69)  3.99% (2.75)  2.29 (1.50-3.50)  118.28 (73.37)  0.25 (0.00-0.50)  29.53 (38.14)  0.50 (0.00-1.00)  13.01 (15.77)  65.6%  12.3%  33.7%  78.6%  5.8% | 6.90 (1.00)  872.77 (71.49)  298.59 (120.66)  501.95 (85.04)  340.80 (84.57)  28.90 (21.32)  1.13 (3.37)  30.03 (22.20)  57.64% (9.25)  38.93% (8.57)  3.30% (2.39)  0.13% (0.39)  3.43% (2.49)  2.50 (1.71-3.43)  121.76 (65.33)  0.29 (0.13-0.57)  29.82 (31.75)  0.33 (0.06-0.85)  10.96 (14.18)  73.7%  13.3%  41.2%  82.7%  7.6% | 6.48 (0.87)  872.67 (74.24)  312.36 (119.68)  550.76 (88.47)  280.92 (72.90)  38.57 (21.34)  2.43 (6.58)  41.00 (23.30)  63.08% (8.45)  32.22% (8.04)  4.43% (2.45)  0.28% (0.74)  4.70% (2.66)  2.86 (2.00-4.00)  138.26 (71.15)  0.29 (0.14-0.57)  31.14 (30.71)  0.71 (0.30-1.40)  18.08 (18.34)  87.2%  28.9%  19.7%  67.7%  7.9% |

| **BMI ≥ 30** | **Total** | **England** | **Norway** | **Portugal** | **Sweden** | |
| --- | --- | --- | --- | --- | --- | --- |
|  |  |  |  |  | *ABC study* | *SNAP study* |
| Mean (SD) number of valid days  Mean (SD) min/day wear time  Mean (SD) activity kcounts/day  Mean (SD) min/day sed time  Mean (SD) min/day light PA  Mean (SD) min/day moderate PA  Mean (SD) min/day vigorous PA  Mean (SD) min/day MVPA  Percentage (SD) sed of wear time  Percentage (SD) light PA of wear time  Percentage (SD) moderate PA of wear time  Percentage (SD) vigorous PA of wear time  Percentage (SD) MVPA of wear time  Median (IQR) number ≥30 min sed bouts  Mean (SD) min/day ≥30 min sed bouts  Median (IQR) number ≥60 min sed bouts  Mean (SD) min/day ≥60 min sed bouts  Median (IQR) number ≥10 min MVPA bouts^a^  Mean (SD) min/day ≥10 min MVPA bouts^a^  Percentage more than 7.5 h/day sed  Percentage more than 10 h/day sed  Percentage not meeting PA recs - based on total time in MVPA  Percentage not meeting PA recs - based on time in ≥10 min MVPA bouts^a^  Percentage more than 10 h/day sed AND not meeting PA recs based on total MVPA | 6.34 (1.19)  861.89 (76.85)  254.63 (114.28)  539.04 (96.59)  297.03 (84.28)  25.12 (20.37)  0.71 (3.53)  25.83 (21.40)  62.57% (9.93)  34.45% (9.36)  2.90% (2.34)  0.08% (0.41)  2.98% (2.46)  3.00 (2.00-4.17)  143.29 (75.74)  0.29 (0.14-0.57)  32.82 (35.58)  0.29 (0.00-0.75)  10.05 (14.65)  83.2%  25.4%  52.2%  85.0%  14.8% | 6.38 (0.95)  847.85 (73.74)  239.67 (117.69)  533.82 (94.14)  291.22 (84.62)  22.43 (20.00)  0.38 (2.35)  22.82 (20.63)  63.05% (10.26)  34.28% (9.32)  2.63% (2.30)  0.04% (0.27)  2.67% (2.37)  3 (2-4.18)  142.51 (77.83)  0.29 (0-0.57)  30.27 (35.01)  0.17 (0-0.67)  7.45 (11.71)  82.6%  22.8%  59.0%  90.5%  16.1% | 6.88 (0.92)  892.82 (74.52)  258.61 (102.79)  570.92 (87.18)  295.47 (78.54)  25.46 (18.54)  0.97 (3.11)  26.43 (19.31)  64.02% (8.58)  33.03% (8.14)  2.84% (2.09)  0.11% (0.34)  2.95% (2.16)  3.29 (2.29-4.44)  153.86 (71.93)  0.33 (0.14-0.67)  34.75 (32.76)  0.43 (0.00-0.86)  12.08 (15.41)  91.4%  35.7%  48.2%  80.9%  19.1% | 4.88 (1.23)  834.06 (79.68)  276.63 (128.41)  486.65 (100.62)  318.77 (92.20)  27.86 (23.90)  0.78 (3.84)  28.64 (24.89)  58.39% (10.99)  38.22% (10.69)  3.30% (2.82)  0.09% (0.46)  3.39% (2.93)  2.75 (1.5-3.71)  127.15 (72.17)  0.25 (0-0.71)  34.04 (38.13)  0.25 (0-0.75)  11.69 (17.71)  68.4%  13.3%  51.5%  81.6%  7.1% | 6.72 (0.93)  865.89 (68.02)  258.25 (98.39)  508.57 (86.92)  335.81 (81.59)  20.96 (17.82)  0.55 (1.89)  21.51 (18.35)  58.78% (9.16)  38.76% (8.76)  2.40% (1.97)  0.06% (0.23)  2.47% (2.04)  2.57 (1.57-3.5)  128.67 (80.85)  0.29 (0.14-0.57)  36.39 (47.09)  0.14 (0-0.43)  6.11 (9.70)  73.7%  14.7%  63.2%  92.6%  11.6% | 6.52 (1.03)  863.88 (69.92)  258.91 (114.87)  560.49 (90.70)  272.11 (75.60)  30.25 (20.59)  1.02 (6.06)  31.27 (23.07)  64.88% (8.99)  31.51% (8.54)  3.50% (2.35)  0.12% (0.72)  3.62% (2.66)  3.17 (2.17-4.29)  149.12 (75.26)  0.29 (0.14-0.57)  32.32 (32.51)  0.43 (0.14-1)  12.81 (16.75)  88.9%  29.6%  38.6%  78.8%  12.7% |

| **EDUCATION - LOWEST** | **Total** | **England** | **Norway** | **Portugal** | **Sweden** | |
| --- | --- | --- | --- | --- | --- | --- |
|  |  |  |  |  | *ABC study* | *SNAP study* |
| Mean (SD) number of valid days  Mean (SD) min/day wear time  Mean (SD) activity kcounts/day  Mean (SD) min/day sed time  Mean (SD) min/day light PA  Mean (SD) min/day moderate PA  Mean (SD) min/day vigorous PA  Mean (SD) min/day MVPA  Percentage (SD) sed of wear time  Percentage (SD) light PA of wear time  Percentage (SD) moderate PA of wear time  Percentage (SD) vigorous PA of wear time  Percentage (SD) MVPA of wear time  Median (IQR) number ≥30 min sed bouts  Mean (SD) min/day ≥30 min sed bouts  Median (IQR) number ≥60 min sed bouts  Mean (SD) min/day ≥60 min sed bouts  Median (IQR) number ≥10 min MVPA bouts^a^  Mean (SD) min/day ≥10 min MVPA bouts^a^  Percentage more than 7.5 h/day sed  Percentage more than 10 h/day sed  Percentage not meeting PA recs - based on total time in MVPA  Percentage not meeting PA recs - based on time in ≥10 min MVPA bouts^a^  Percentage more than 10 h/day sed AND not meeting PA recs based on total MVPA | 6.40 (1.18)  854.44 (78.54)  277.46 (142.26)  511.90 (97.00)  313.76 (92.03)  27.92 (25.01)  0.86 (4.40)  28.78 (26.23)  60.05% (10.81)  36.62% (9.94)  3.23% (2.74)  0.10% (0.52)  3.33% (2.89)  2.71 (1.75-3.75)  131.33 (75.69)  0.29 (0.13-0.56)  29.93 (35.18)  0.29 (0.00-0.86)  11.78 (17.21)  75.7%  18.2%  48.0%  81.1%  12.0% | 6.52 (0.84)  842.83 (72.99)  247.13 (129.44)  522.22 (92.18)  296.64 (89.12)  23.31 (21.89)  0.65 (3.40)  23.96 (22.78)  62.14% (10.59)  35.05% (9.59)  2.74% (2.52)  0.08% (0.40)  2.82% (2.62)  3.00 (1.96-4.14)  140.72 (77.85)  0.29 (0.14-0.57)  29.67 (33.18)  0.20 (0.00-0.71)  8.68 (13.72)  79.0%  19.4%  57.6%  86.5%  15.9% | 6.85 (0.99)  876.77 (76.73)  285.46 (140.76)  536.28 (83.18)  310.01 (86.52)  29.32 (24.65)  1.16 (5.94)  30.48 (26.80)  61.39% (9.32)  35.16% (8.59)  3.33% (2.83)  0.13% (0.69)  3.46% (3.08)  2.86 (2.00-4.00)  139.62 (75.96)  0.29 (0.14-0.57)  33.29 (36.87)  0.43 (0.00-1.00)  15.58 (22.33)  85.8%  22.6%  44.4%  76.1%  13.4% | 4.55 (0.89)  823.02 (75.24)  304.30 (155.54)  443.66 (110.20)  349.45 (107.94)  29.67 (25.39)  0.24 (1.32)  29.91 (25.77)  54.10% (13.43)  42.30% (12.15)  3.57% (3.03)  0.03% (0.15)  3.60% (3.07)  2.20 (1.25-3.25)  110.38 (79.87)  0.25 (0.00-0.50)  30.59 (45.00)  0.27 (0.00-1.00)  10.27 (13.85)  51.0%  9.9%  47.5%  83.7%  7.9% | 6.88 (1.02)  855.04 (69.82)  279.16 (118.84)  486.97 (85.37)  341.91 (88.98)  25.61 (20.24)  0.56 (2.28)  26.17 (20.74)  57.14% (9.97)  39.83% (9.18)  2.97% (2.28)  0.06% (0.27)  3.03% (2.33)  2.43 (1.67-3.50)  124.20 (71.65)  0.29 (0.13-0.57)  31.69 (36.61)  0.29 (0.00-0.71)  9.68 (12.26)  65.1%  9.3%  52.1%  84.2%  6.5% | 6.52 (0.99)  869.78 (89.23)  305.45 (165.69)  531.28 (95.01)  300.69 (80.53)  36.20 (32.16)  1.61 (6.14)  37.81 (33.57)  61.12% (9.32)  34.59% (8.76)  4.09% (2.91)  0.19% (0.73)  4.28% (3.13)  2.57 (1.75-3.43)  122.10 (64.98)  0.29 (0.14-0.43)  22.93 (22.85)  0.57 (0.14-1.23)  15.54 (19.11)  82.7%  23.6%  29.5%  72.6%  9.7% |

| **EDUCATION – SECOND LOWEST** | **Total** | **England** | **Norway** | **Portugal** | **Sweden** | |
| --- | --- | --- | --- | --- | --- | --- |
|  |  |  |  |  | *ABC study* | *SNAP study* |
| Mean (SD) number of valid days  Mean (SD) min/day wear time  Mean (SD) activity kcounts/day  Mean (SD) min/day sed time  Mean (SD) min/day light PA  Mean (SD) min/day moderate PA  Mean (SD) min/day vigorous PA  Mean (SD) min/day MVPA  Percentage (SD) sed of wear time  Percentage (SD) light PA of wear time  Percentage (SD) moderate PA of wear time  Percentage (SD) vigorous PA of wear time  Percentage (SD) MVPA of wear time  Median (IQR) number ≥30 min sed bouts  Mean (SD) min/day ≥30 min sed bouts  Median (IQR) number ≥60 min sed bouts  Mean (SD) min/day ≥60 min sed bouts  Median (IQR) number ≥10 min MVPA bouts^a^  Mean (SD) min/day ≥10 min MVPA bouts^a^  Percentage more than 7.5 h/day sed  Percentage more than 10 h/day sed  Percentage not meeting PA recs - based on total time in MVPA  Percentage not meeting PA recs - based on time in ≥10 min MVPA bouts^a^  Percentage more than 10 h/day sed AND not meeting PA recs based on total MVPA | 6.57 (1.08)  874.17 (72.16)  309.88 (128.35)  518.27 (92.71)  321.75 (84.32)  32.46 (22.00)  1.69 (5.38)  34.15 (23.79)  59.32% (9.65)  36.79% (9.09)  3.70% (2.48)  0.19% (0.59)  3.89% (2.67)  2.57 (1.67-3.63)  123.78 (70.30)  0.25 (0.00-0.50)  27.54 (31.17)  0.50 (0.14-1.14)  14.73 (17.46)  77.2%  19.0%  34.7%  74.0%  8.3% | 6.39 (0.90)  845.10 (72.84)  289.17 (127.09)  501.02 (92.45)  312.96 (82.72)  30.12 (22.97)  0.99 (3.27)  31.11 (24.02)  59.33% (10.05)  37.00% (9.13)  3.55% (2.67)  0.12% (0.39)  3.67% (2.80)  2.43 (1.57-3.50)  119.17 (73.36)  0.17 (0.00-0.43)  24.44 (30.84)  0.40 (0.00-1.00)  11.56 (15.19)  71.3%  14.2%  43.1%  81.2%  8.6% | 6.88 (0.93)  890.93 (67.69)  308.13 (127.52)  541.19 (83.35)  315.76 (79.39)  32.04 (21.42)  1.94 (5.83)  33.98 (23.59)  60.83% (8.72)  35.37% (8.20)  3.59% (2.37)  0.21% (0.63)  3.80% (2.59)  2.71 (1.86-3.85)  132.45 (69.67)  0.29 (0.14-0.57)  30.77 (31.64)  0.57 (0.14-1.14)  16.25 (18.30)  85.9%  23.4%  34.5%  70.9%  10.2% | 4.57 (1.01)  838.84 (67.26)  326.32 (120.56)  448.52 (95.97)  356.29 (92.22)  32.93 (21.48)  1.10 (4.20)  34.03 (22.53)  53.47% (10.60)  42.49% (10.47)  3.90% (2.51)  0.13% (0.51)  4.04% (2.64)  2.00 (1.11-2.76)  98.02 (66.26)  0.25 (000-0.50)  22.38 (26.99)  0.50 (0.00-1.00)  12.28 (16.77)  52.0%  6.0%  34.0%  78.7%  1.3% | 6.78 (1.09)  878.11 (71.01)  320.60 (128.99)  480.56 (92)  366.64 (88.94)  29.62 (21.07)  1.29 (4.16)  30.91 (22.38)  54.81% (9.89)  41.69% (9.21)  3.36% (2.31)  0.14% (0.46)  3.50% (2.45)  2.17 (1.43-3.16)  108.36 (65.10)  0.17 (0.00-0.43)  25.42 (33.67)  0.35 (0.00-0.75)  10.32 (13.15)  62.7%  12.3%  38.9%  82.8%  7.4% | 6.51 (0.78)  872.84 (69.78)  333.49 (131.58)  524.36 (93.65)  306.15 (81.64)  39.81 (22.04)  2.53 (7.22)  42.33 (24.07)  60.07% (9.53)  35.09% (8.96)  4.56% (2.52)  0.28% (0.77)  4.85% (2.74)  2.67 (1.71-3.71)  123.60 (67.97)  0.25 (0.00-0.43)  24.74 (28.58)  0.83 (0.29-1.40)  18.82 (19.30)  78.4%  21.9%  19.0%  64.8%  4.8% |

| **EDUCATION – SECOND HIGHEST** | **Total** | **England** | **Norway** | **Portugal** | **Sweden** | |
| --- | --- | --- | --- | --- | --- | --- |
|  |  |  |  |  | *ABC study* | *SNAP study* |
| Mean (SD) number of valid days  Mean (SD) min/day wear time  Mean (SD) activity kcounts/day  Mean (SD) min/day sed time  Mean (SD) min/day light PA  Mean (SD) min/day moderate PA  Mean (SD) min/day vigorous PA  Mean (SD) min/day MVPA  Percentage (SD) sed of wear time  Percentage (SD) light PA of wear time  Percentage (SD) moderate PA of wear time  Percentage (SD) vigorous PA of wear time  Percentage (SD) MVPA of wear time  Median (IQR) number ≥30 min sed bouts  Mean (SD) min/day ≥30 min sed bouts  Median (IQR) number ≥60 min sed bouts  Mean (SD) min/day ≥60 min sed bouts  Median (IQR) number ≥10 min MVPA bouts^a^  Mean (SD) min/day ≥10 min MVPA bouts^a^  Percentage more than 7.5 h/day sed  Percentage more than 10 h/day sed  Percentage not meeting PA recs - based on total time in MVPA  Percentage not meeting PA recs - based on time in ≥10 min MVPA bouts^a^  Percentage more than 10 h/day sed AND not meeting PA recs based on total MVPA | 6.50 (1.15)  872.57 (71.29)  306.72 (121.07)  537.61 (91.55)  298.34 (80.82)  34.27 (21.79)  2.35 (5.66)  36.62 (23.77)  61.61% (9.17)  34.20% (8.84)  3.93% (2.49)  0.27% (0.65)  4.20% (2.71)  2.75 (1.86-3.83)  131.80 (71.08)  0.29 (0.14-0.57)  30.25 (34.04)  0.60 (0.17-1.18)  16.60 (18.15)  82.9%  25.2%  30.3%  70.4%  9.5% | 6.45 (0.86)  850.75 (70.45)  283.08 (122.77)  512.90 (89.53)  307.39 (79.04)  29.30 (22.81)  1.17 (4.40)  30.46 (24.23)  60.31% (9.42)  36.14% (8.83)  3.42% (2.57)  0.14% (0.50)  3.55% (2.73)  2.50 (1.57-3.55)  118.87 (70.65)  0.20 (0.00-0.43)  24.54 (30.74)  0.40 (0.14-1.00)  12.28 (17.19)  77.0%  14.9%  45.1%  81.2%  8.4% | 6.89 (0.91)  891.98 (65.74)  298.87 (110.94)  562.61 (83.27)  294.23 (77.33)  32.85 (20.10)  2.29 (4.81)  35.14 (21.54)  63.13% (8.46)  32.94% (8.13)  3.68% (2.25)  0.26% (0.55)  3.94% (2.42)  3.00 (2.14-4.00)  142.66 (70.35)  0.33 (0.14-0.57)  33.80 (32.36)  0.67 (0.25-1.14)  18.27 (18.33)  90.8%  33.3%  30.5%  66.9%  13.8% | 4.39 (0.85)  850.70 (76.51)  306.70 (126.60)  501.70 (95.05)  314.17 (92.37)  33.12 (22.65)  1.71 (5.84)  34.83 (24.78)  59.06% (10.09)  36.84% (9.86)  3.91% (2.68)  0.19% (0.63)  4.10% (2.87)  2.50 (1.55-3.63)  121.00 (68.51)  0.25 (0.00-0.50)  26.69 (31.62)  0.50 (0.00-1.00)  13.66 (18.07)  71.1%  15.4%  34.2%  77.9%  6.7% | 6.76 (1.37)  869.83 (73.68)  323.29 (127.81)  501.09 (98.57)  333.38 (86.92)  33.11 (21.55)  2.26 (5.03)  35.37 (23.57)  57.60% (10.00)  38.34% (9.35)  3.80% (2.43)  0.26% (0.59)  4.06% (2.68)  2.29 (1.31-3.31)  114.20 (76.05)  0.25 (0.00-0.50)  29.72 (49.00)  0.50 (0.14-1.00)  12.08 (13.30)  70.4%  16.2%  32.8%  79.1%  8.3% | 6.45 (0.89)  865.77 (69.87)  323.16 (125.62)  544.51 (87.16)  278.06 (73.11)  39.89 (22.22)  3.31 (7.24)  43.20 (24.83)  62.84% (8.50)  32.15% (8.18)  4.62% (2.58)  0.38% (0.84)  5.01% (2.88)  2.86 (2.00-4.00)  135.52 (67.64)  0.29 (0.14-0.57)  29.90 (29.43)  0.75 (0.29-1.57)  19.71 (19.42)  84.5%  27.0%  19.4%  62.8%  5.5% |

| **EDUCATION - HIGHEST** | **Total** | **England** | **Norway** | **Portugal** | **Sweden** | |
| --- | --- | --- | --- | --- | --- | --- |
|  |  |  |  |  | *ABC study* | *SNAP study* |
| Mean (SD) number of valid days  Mean (SD) min/day wear time  Mean (SD) activity kcounts/day  Mean (SD) min/day sed time  Mean (SD) min/day light PA  Mean (SD) min/day moderate PA  Mean (SD) min/day vigorous PA  Mean (SD) min/day MVPA  Percentage (SD) sed of wear time  Percentage (SD) light PA of wear time  Percentage (SD) moderate PA of wear time  Percentage (SD) vigorous PA of wear time  Percentage (SD) MVPA of wear time  Median (IQR) number ≥30 min sed bouts  Mean (SD) min/day ≥30 min sed bouts  Median (IQR) number ≥60 min sed bouts  Mean (SD) min/day ≥60 min sed bouts  Median (IQR) number ≥10 min MVPA bouts^a^  Mean (SD) min/day ≥10 min MVPA bouts^a^  Percentage more than 7.5 h/day sed  Percentage more than 10 h/day sed  Percentage not meeting PA recs - based on total time in MVPA  Percentage not meeting PA recs - based on time in ≥10 min MVPA bouts^a^  Percentage more than 10 h/day sed AND not meeting PA recs based on total MVPA | 6.42 (1.08)  875.15 (67.58)  317.93 (120.38)  546.78 (85.75)  288.53 (75.72)  36.75 (21.04)  3.08 (6.51)  39.84 (23.19)  62.48% (8.56)  32.96% (8.26)  4.21% (2.40)  0.35% (0.75)  4.56% (2.65)  3.00 (2.00-4.00)  141.62 (73.50)  0.33 (0.14-0.60)  34.76 (35.39)  0.71 (0.29-1.4)  18.53 (18.68)  86.5%  27.7%  22.0%  66.7%  7.6% | 6.43 (0.88)  859.73 (69.78)  289.72 (131.36)  540.42 (87.39)  285.55 (78.69)  31.67 (23.81)  2.09 (5.19)  33.76 (25.46)  62.92% (9.16)  33.17% (8.54)  3.68% (2.72)  0.24% (0.58)  3.92% (2.90)  3.00 (1.86-4.14)  139.17 (73.89)  0.29 (0.14-0.60)  33.14 (32.44)  0.57 (0.14-1.14)  15.10 (20.11)  84.0%  25.6%  36.0%  75.8%  11.3% | 6.83 (0.83)  898.37 (58.53)  320.00 (118.71)  568.92 (78.55)  289.94 (73.44)  35.86 (20.09)  3.65 (7.09)  39.51 (22.57)  63.36% (8.01)  32.24% (7.79)  3.99% (2.23)  0.41% (0.79)  4.40% (2.51)  3.00 (2.00-4.17)  146.51 (73.78)  0.33 (0.14-0.67)  37.23 (35.97)  0.75 (0.34-1.32)  20.62 (19.6)  92.1%  36.9%  20.4%  62.5%  9.7% | 4.44 (0.88)  845.84 (72.94)  300.79 (114.23)  508.74 (89.28)  302.62 (79.23)  32.04 (19.35)  2.43 (5.71)  34.47 (21.52)  60.15% (9.07)  35.77% (8.76)  3.79% (2.30)  0.29% (0.68)  4.08% (2.54)  2.75 (1.80-3.75)  130.38 (66.07)  0.25 (0.00-0.50)  31.26 (32.67)  0.50 (0.00-1.00)  12.72 (15.35)  75.0%  16.4%  32.2%  79.5%  6.2% | 6.84 (0.87)  876.76 (64.63)  324.15 (120.64)  513.57 (87.32)  326.79 (81.23)  34.08 (20.90)  2.32 (4.82)  36.40 (22.50)  58.60% (9.07)  37.24% (8.68)  3.89% (2.34)  0.26% (0.55)  4.16% (2.53)  2.57 (1.71-3.50)  125.47 (74.23)  0.29 (0.14-0.57)  34.02 (41.03)  0.57 (0.17-1.00)  14.91 (16.93)  76.7%  16.0%  27.1%  74.1%  6.1% | 6.51 (0.88)  872.54 (66.99)  329.43 (116.41)  552.77 (82.56)  275.30 (69.45)  41.02 (20.10)  3.45 (7.05)  44.47 (22.26)  63.34% (8.02)  31.55% (7.65)  4.72% (2.32)  0.40% (0.83)  5.11% (2.58)  3.14 (2.14-4.14)  146.35 (73.75)  0.33 (0.14-0.60)  34.81 (35.00)  0.93 (0.43-1.57)  20.80 (18.05)  89.1%  28.3%  14.1%  61.0%  5.5% |

ABC = Attitude and Behaviour Change; SNAP = Swedish Neighborhood and Physical Activity; SD = standard deviation; min = minutes; kcounts = kilocounts; sed = sedentary; PA = physical activity; MVPA = moderate-to-vigorous physical activity; IQR = interquartile range; h = hours; recs = recommendations. a.With allowance for interruptions of max 2 minutes below threshold.

Table S2. Distribution and multivariable odds ratio of accumulating more than 7.5 hours/day of sedentary time by gender, age, BMI and educational level. Results are shown for the total sample and the different study samples.

|  | **Total** | | **England** | | **Norway** | | **Portugal** | | **Sweden** | | | |
| --- | --- | --- | --- | --- | --- | --- | --- | --- | --- | --- | --- | --- |
|  |  |  |  |  |  |  |  |  | *ABC study* | | *SNAP study* | |
|  | % | OR (95% CI)^*^ | % | OR (95% CI)^**^ | % | OR (95% CI)^**^ | % | OR (95% CI)^**^ | % | OR (95% CI)^**^ | % | OR (95% CI)^**^ |
| **Overall** | 81.1 | N/A | 77.8 | N/A | 88.7 | N/A | 66.7 | N/A | 69.5 | N/A | 86.0 | N/A |
| **Gender**  Men (ref)  Women | 85.2  77.8 | 1.00  **0.62 (0.55-0.70)** | 81.0  75.2 | 1.00  0.89 (0.69-1.14) | 91.6  86.1 | 1.00  **0.67 (0.53-0.86)** | 74.7  61.9 | 1.00  **0.48 (0.34-0.68)** | 71.9  67.6 | 1.00  0.93 (0.69-1.24) | 90.1  82.7 | 1.00  **0.57 (0.43-0.75)** |
| **Age, years**  20-35 (ref)  36 – 50  51 – 66  67 – 75 | 78.9  79.7  83.8  81.2 | 1.00  1.05 (0.91-1.22)  **1.57 (1.33-1.85)**  **2.38 (1.86-3.05)** | 72.9  69.0  81.5  90.1 | 1.00  0.87 (0.62-1.23)  **1.61 (1.11-2.33)**  **4.54 (2.70-7.63)** | 87.4  88.8  89.3  88.9 | 1.00  0.96 (0.69-1.33)  1.14 (0.82-1.59)  **1.83 (1.13-2.97)** | 73.3  64.8  63.1  64.8 | 1.00  0.85 (0.55-1.31)  1.68 (0.95-2.96)  **4.46 (2.28-8.73)** | 68.4  61.6  76.1  76.8 | 1.00  **0.63 (0.44-0.91)**  1.48 (0.99-2.22)  **2.09 (1.14-3.85)** | 81.9  86.3  87.8  N/A | 1.00  1.15 (0.82-1.61)  **1.57 (1.10-2.23)**  N/A |
| **Weight status**  BMI < 25 (ref)  BMI 25 – 30  BMI ≥ 30 | 80.4  81.8  83.2 | 1.00  **1.18 (1.04-1.34)**  **1.56 (1.30-1.88)** | 73.0  78.0  82.6 | 1.00  1.19 (0.89-1.59)  **1.81 (1.29-2.55)** | 87.4  89.8  91.4 | 1.00  1.25 (0.96-1.63)  **1.72 (1.13-2.60)** | 67.4  65.6  68.4 | 1.00  0.90 (0.62-1.33)  1.42 (0.84-2.37) | 66.8  73.7  73.7 | 1.00  **1.49 (1.08-2.05)**  **1.72 (1.01-2.93)** | 85.0  87.2  88.9 | 1.00  1.16 (0.86-1.56)  **1.70 (1.01-2.85)** |
| **Education**  Lowest (ref)  Second lowest  Second highest  Highest | 75.7  77.2  82.9  86.5 | 1.00  1.08 (0.91-1.28)  **1.85 (1.53-2.24)**  **2.71 (2.26-3.26)** | 79.0  71.3  76.9  84.0 | 1.00  0.94 (0.67-1.34)  1.44 (0.96-2.17)  **2.30 (1.55-3.43)** | 85.8  85.9  90.8  92.1 | 1.00  1.00 (0.69-1.43)  **1.69 (1.12-2.57)**  **2.07 (1.35-3.20)** | 51.0  52.0  71.1  75.0 | 1.00  1.69 (0.99-2.90)  **4.05 (2.19-7.50)**  **6.04 (3.33-10.95)** | 65.1  62.7  70.4  76.7 | 1.00  1.03 (0.67-1.59)  **1.81 (1.14-2.88)**  **2.31 (1.50-3.55)** | 82.7  78.4  84.5  89.1 | 1.00  0.64 (0.39-1.04)  1.28 (0.80-2.05)  **1.80 (1.18-2.76)** |

ABC = Attitude and Behaviour Change study; SNAP = Swedish Neighborhood and Physical Activity study. Numbers in bold represent p < 0.05. *Adjusted for gender, age, BMI, educational level, study and wear time. **Adjusted for gender, age, BMI, educational level and wear time.

Table S3. Distribution and multivariable odds ratio of not meeting physical activity recommendations based on accumulating more than 150 min/week of MVPA in ≥ 10 min MVPA bouts^b^ by gender, age, BMI and educational level. Results are shown for the total sample and the different study samples.

|  | **Total** | | **England** | | **Norway** | | **Portugal** | | **Sweden** | | | |
| --- | --- | --- | --- | --- | --- | --- | --- | --- | --- | --- | --- | --- |
|  |  |  |  |  |  |  |  |  | *ABC study* | | *SNAP study* | |
|  | % | OR (95% CI)^*^ | % | OR (95% CI)^**^ | % | OR (95% CI)^**^ | % | OR (95% CI)^**^ | % | OR (95% CI)^**^ | % | OR (95% CI)^**^ |
| **Overall** | 72.1 | N/A | 81.3 | N/A | 68.4 | N/A | 79.4 | N/A | 79.3 | N/A | 63.1 | N/A |
| **Gender**  Men (ref)  Women | 72.3  71.9 | 1.00  1.01 (0.92-1.12) | 77.7  84.3 | 1.00  **1.57 (1.21-2.04)** | 71.7  65.5 | 1.00  **0.80 (0.68-0.94)** | 76.5  81.1 | 1.00  **1.47 (1.02-2.11)** | 80.2  78.6 | 1.00  0.96 (0.70-1.32) | 63.8  62.6 | 1.00  1.00 (0.84-1.19) |
| **Age, years**  20-35 (ref)  36 – 50  51 – 66  67 – 75 | 72.1  71.9  69.8  80.5 | 1.00  1.00 (0.88-1.14)  **0.75 (0.66-0.86)**  0.87 (0.70-1.08) | 76.4  77.5  82.1  91.5 | 1.00  1.06 (0.75-1.52)  1.09 (0.76-1.56)  **2.59 (1.5-4.46)** | 71.4  70.9  64.5  66.6 | 1.00  0.88 (0.71-1.11)  **0.59 (0.47-0.74)**  **0.56 (0.41-0.78)** | 79.9  76.2  75.7  86.3 | 1.00  0.90 (0.56-1.45)  **0.51 (0.29-0.89)**  0.89 (0.44-1.80) | 76.8  82.3  80.5  72.6 | 1.00  1.44 (0.95-2.17)  1.05 (0.70-1.59)  0.64 (0.36-1.15) | 62.0  65.1  61.7  N/A | 1.00  1.06 (0.84-1.34)  0.84 (0.66-1.07)  N/A |
| **Weight status**  BMI < 25 (ref)  BMI 25 – 30  BMI ≥ 30 | 65.6  74.8  84.9 | 1.00  **1.47 (1.32-1.64)**  **2.66 (2.23-3.18)** | 74.7  80.0  90.5 | 1.00  **1.41 (1.06-1.87)**  **3.16 (2.15-4.64)** | 62.2  72.0  80.9 | 1.00  **1.51 (1.27-1.79)**  **2.41 (1.81-3.21)** | 78.1  78.6  81.6 | 1.00  1.24 (0.82-1.87)  1.64 (0.90-3.00) | 74.5  82.7  92.6 | 1.00  **1.52 (1.07-2.15)**  **4.03 (1.81-8.97)** | 58.5  67.7  78.8 | 1.00  **1.48 (1.21-1.80)**  **2.65 (1.83-3.83)** |
| **Education**  Lowest (ref)  Second lowest  Second highest  Highest | 81.1  74.0  70.4  66.7 | 1.00  **0.71 (0.60-0.84)**  **0.64 (0.53-0.76)**  **0.57 (0.49-0.68)** | 86.5  81.2  81.2  75.8 | 1.00  0.78 (0.53-1.15)  0.88 (0.57-1.34)  0.75 (0.51-1.11) | 76.1  70.9  66.8  62.5 | 1.00  **0.67 (0.51-0.89)**  **0.58 (0.43-0.78)**  **0.47 (0.35-0.63)** | 83.7  78.7  77.9  79.5 | 1.00  0.72 (0.39-1.32)  0.67 (0.35-1.28)  0.75 (0.40-1.41) | 84.2  82.8  79.1  74.1 | 1.00  0.85 (0.50-1.45)  0.74 (0.44-1.25)  **0.55 (0.34-0.89)** | 72.6  64.8  62.8  61.0 | 1.00  **0.63 (0.43-0.92)**  **0.62 (0.43-0.87)**  **0.59 (0.43-0.81)** |

ABC = Attitude and Behaviour Change study; SNAP = Swedish Neighborhood and Physical Activity study. Numbers in bold represent p < 0.05. *Adjusted for gender, age, BMI, educational level, study and wear time. **Adjusted for gender, age, BMI, educational level and wear time.
